# The stress hyperglycemia ratio as a predictor of short- and long-term mortality in patients with acute brain injury: a retrospective cohort study

**DOI:** 10.3389/fneur.2025.1552462

**Published:** 2025-04-28

**Authors:** Juan Wang, Peng-fei Ding, Zheng Peng, Chun-Hua Hang, Wei Li

**Affiliations:** ^1^Department of Neurosurgery, Nanjing Drum Tower Hospital, Clinical College of Nanjing University of Chinese Medicine, Nanjing, China; ^2^Department of Neurosurgery, Nanjing Drum Tower Hospital, Clinical College of Nanjing Medical University, Nanjing, China; ^3^Neurosurgical Institute, Nanjing University, Nanjing, China

**Keywords:** acute brain injury, stress hyperglycemia ratio (SHR), linear association, short-term and long-term mortality, ROC curve

## Abstract

**Background:**

This study examines the Stress Hyperglycemia Ratio (SHR) as a predictor of mortality in acute brain injury (ABI) patients using the MIMIC-IV v3. 1 database.

**Methods:**

In this retrospective cohort study of 2,423 ABI patients, SHR was calculated as SHR = [Admission blood glucose (mg/dL)] / [28.7 × HbA1c (%) – 46.7]. Mortality outcomes included ICU, in-hospital, 30, 60, 90, and 365-day mortality. Cox regression models adjusted for covariates assessed the association between SHR and mortality risk, with restricted cubic splines confirming linearity. Predictive performance was evaluated using ROC curves, incorporating SHR, Glasgow Coma Scale (GCS), and first-day ventilation status.

**Results:**

SHR was significantly associated with mortality across all outcomes, showing a linear relationship. Adjusted hazard ratios (HR) for in-hospital and ICU mortality were 1.18 (95% CI: 1.06–1.32, *p* = 0.003) and 1.16 (95% CI: 1.02–1.32, *p* = 0.029), respectively. Dichotomized SHR indicated increased in-hospital mortality risk (HR: 1.44, 95% CI: 1.13–1.83, *p* = 0.003). Combining SHR with GCS and ventilation status improved predictive accuracy, achieving AUCs of 0.817 for ICU mortality and 0.788 for in-hospital mortality. Robustness was supported by E-values of 2.24 and 2.37 for in-hospital and ICU mortality.

**Conclusion:**

SHR independently predicts short- and long-term mortality in ABI patients, with enhanced utility when combined with GCS and ventilation status, supporting its role in clinical risk stratification.

## Introduction

Acute brain injury (ABI), including traumatic brain injury (TBI), intracerebral hemorrhage (ICH), and ischemic stroke, is a critical condition characterized by high mortality and prolonged recovery, posing significant challenges in critical care (1). The neuroendocrine stress response in ABI often results in transient hyperglycemia. Unlike chronic hyperglycemia, stress-induced hyperglycemia reflects an immediate response to injury and is associated with poorer outcomes in critically ill patients (2). Traditional measures, such as admission blood glucose (ABG), have limited predictive value as they do not distinguish baseline glycemic control from acute hyperglycemia, especially in patients with diabetes.

To address these limitations, the stress hyperglycemia ratio (SHR) has been introduced as a specific indicator of hyperglycemia resulting from physiological stress. SHR is calculated by adjusting acute blood glucose levels relative to baseline glycemic control, specifically taking into account both the patient's current glucose levels and long-term glycemic status (as indicated by HbA1c) (2, 3). Our preliminary work suggested a U-shaped relationship between glucose variability and all-cause mortality in ABI patients, with significant interactions involving age and diabetes status, indicating the potential value of a reliable predictor that can capture the impact of hyperglycemia on ABI prognosis.

This study investigates SHR as a predictor of primary outcomes (in-hospital, ICU, and 365-day mortality) and secondary outcomes (30, 60, and 90-day mortality) in ABI patients. Recognizing the Glasgow Coma Scale (GCS) as an established neurological assessment in neurosurgical populations and the significant impact of mechanical ventilation on ABI prognosis, we further evaluate the predictive power of SHR in conjunction with GCS scores and ICU ventilation on day one. By analyzing SHR alongside these clinical parameters, we aim to enhance risk stratification in ABI and offer insights for future management strategies.

## Methods

### Database source

This study utilized data from the Medical Information Mart for Intensive Care version 3.1 (MIMIC-IV v3.1), a comprehensive clinical dataset from the Beth Israel Deaconess Medical Center in Boston, Massachusetts, covering the period from 2008 to 2022. The MIMIC-IV v3.1 database contains 94,458 admissions, providing detailed clinical information on demographics, vital signs, laboratory results, comorbidities, treatments, and discharge outcomes. Renowned for its rigor and depth, MIMIC-IV v3.1 is extensively used in clinical research, particularly for critical care data. Access to this publicly available dataset was granted with ethical approval, with author Juan Wang certified to utilize the data (certification number: 13313422). All analyses adhered to the Strengthening the Reporting of Observational Studies in Epidemiology (STROBE) guidelines to ensure transparency and reproducibility (4).

### Data collection

#### Inclusion and exclusion criteria

Inclusion criteria were first ICU admission during initial hospitalization to ensure unique patient records, age ≥18 years, documented diagnosis of ABI including traumatic brain injury, spontaneous intracranial hemorrhage, and ischemic stroke, and availability of both glucose and glycated hemoglobin (HbA1c) measurements for calculating the SHR. Exclusion criteria included missing glucose or HbA1c data, ICU stays of <24 h, and ABI resulting from secondary etiologies such as tumors, infections, metabolic disorders, or toxic causes. After applying these criteria, 2,423 patients were included in the final analysis.

#### Data extraction and definitions

Data were extracted from the MIMIC-IV v3.1 database using Navicat Premium (version 17) and SQL queries. Variables were categorized as follows, based on established methodologies in the MIMIC database and SHR research (3, 5, 6). These variables are detailed in Supplementary Table 1. Demographic: age, sex, and weight; Vital signs: baseline measurements recorded within the first 24 h of ICU admission, including heart rate (HR, beats per minute), mean blood pressure (MBP, mmHg), respiratory rate (RR, breaths per minute), temperature (°C), and oxygen saturation (SpO_2_, %); Laboratory tests: hemoglobin (g/dL), platelets (× 10^9^/L), red blood cell count (RBC, × 10^12^/L), white blood cell count (WBC, × 10^9^/L), blood urea nitrogen (BUN, mg/dL), creatinine (mg/dL), sodium (mmol/L), potassium (mmol/L), and aspartate aminotransferase (AST, U/L);Medical history and comorbidities: smoking status and conditions such as dementia, Cerebrovascular disease (CBD), cancer, rheumatic disease, liver disease, hyperlipidemia, diabetes, hypertension, myocardial infarction (MI), congestive heart failure (CHF), and sepsis, as defined by Sepsis-3 criteria; Organ dysfunction and severity: Charlson Comorbidity Index (CCI), Glasgow Coma Scale (GCS), and Simplified Acute Physiology Score II (SAPS II);In-hospital procedures: mechanical ventilation on the first ICU day, craniotomy, percutaneous cerebral arterial embolization (Pe), ventricular drainage (Vd), and the use of diuretics and β-blockers.

#### Exposure definition

The SHR was calculated to quantify stress-induced hyperglycemia, adjusting for baseline glycemic control. The formula used was SHR = [Admission blood glucose (mg/dL)] / [28.7 × HbA1c (%) – 46.7]. This formula is commonly applied in critical care research, as it standardizes the assessment of acute hyperglycemia by considering both current and chronic glucose levels (3, 7).

#### Outcome measures

The primary outcomes of this study included in-hospital and ICU mortality as short-term indicators, and 365-day mortality as a long-term indicator. Additionally, 30, 60, and 90-day mortality were assessed as secondary short-term outcomes. These outcomes collectively provide a comprehensive evaluation of both short- and long-term mortality risks (8, 9).

### Statistical analyses

Statistical analyses were performed using R Statistical Software (version 4.2.2) and the Free Statistics analysis platform (version 2.0, Beijing, China). Cox regression analysis was conducted using the coxph function from the survival package, and ROC curves were generated using the roc function from the pROC package in R. The Kolmogorov-Smirnov test assessed the normality of continuous variables. Normally distributed continuous variables were reported as mean ± standard deviation (SD), while non-normally distributed variables were reported as median and interquartile range (IQR). Categorical variables were summarized as frequencies and percentages. Group comparisons used the independent samples *t*-test or Mann-Whitney U-test for continuous variables, and the chi-square or Fisher's exact test for categorical variables. Bonferroni adjustments were applied where necessary to reduce Type I error due to multiple comparisons, with statistical significance set at a two-sided *p-*value <0.05.

Baseline characteristics for the two exposure groups (high and low SHR) were presented before and after imputation ensuring robust comparisons across exposure categories.

Cox proportional hazards regression models were used to evaluate the association between SHR and mortality outcomes, including in-hospital, ICU, 365, 30, 60, and 90-day mortality. SHR was analyzed as a continuous variable and dichotomized to assess mortality risk across SHR levels. The proportional hazards assumption was tested with log-log survival plots and Schoenfeld residuals. Kaplan-Meier survival curves were generated for SHR categories to depict survival probabilities, with statistical differences across groups assessed via the log-rank test.

To account for potential confounders, three progressively adjusted Cox regression models were developed: (1) Model 1: unadjusted; (2) Model 2: adjusted for demographic and clinical variables; (3) Model 3: further adjusted for additional covariates, including Clinical Severity Scores and In-hospital Procedures. These variables were selected based on univariate analysis (*p* < 0.1) and their clinical relevance. Additionally, stepwise regression analysis, as part of a sensitivity analysis, identified the final set of predictors for mortality outcomes, which were incorporated into the models.

Receiver operating characteristic (ROC) curves were generated to evaluate the predictive performance of SHR for mortality outcomes, assessed both independently and in combination with GCS scores and mechanical ventilation on the first ICU-day (10, 11). As part of a sensitivity analysis, LASSO regression was conducted to perform variable selection and further validate the predictive value of SHR for mortality outcomes. The area under the curve (AUC) was calculated to compare the predictive power of models for short- and long-term mortality, covering in-hospital, ICU, 30, 60, 90, and 365-day mortality. For each model, performance metrics including specificity, sensitivity, accuracy, precision, and recall were reported.

Subgroup analyses explored potential effect modifications by variables such as age, hypertension, diabetes, sepsis, and craniotomy status, using interaction terms with SHR to evaluate group heterogeneity. To examine the consistency of the association between SHR and mortality outcomes, we categorized SHR as a continuous, dichotomized, variable, with dichotomization based on the median value of SHR observed in our cohort, and restricted cubic splines confirmed the linear association between SHR and mortality outcomes.

To ensure robustness, the primary and secondary outcomes of this study, including in-hospital, ICU, and 365-day mortality as long-term indicators, and 30, 60, and 90-day mortality for short-term assessment, were analyzed. Sevenfold multiple imputation applied to address missing data using the “mice” package in R. *E*-values were calculated to estimate the minimum strength of association required for an unmeasured confounder to explain the observed association between SHR and mortality outcomes. The *E-*value represents the smallest effect size that an unmeasured confounder would need to havein order to fully account for the observed relationship.

## Results

Supplementary Figure 1 presents the screening process of 94,458 ICU admissions from the MIMIC-IV v3.1 database. After applying inclusion criteria to select only first ICU admissions, 6,824 patients with complete data for SHR calculation were identified. Additional exclusions based on age, ICU stay duration, and ABI diagnosis resulted in a final cohort of 2,423 patients.

### Cohort characteristics

Baseline characteristics of the cohort are summarized in Table 1, with patients categorized into two SHR groups: Group 1 (SHR 0.185–1.021) and Group 2 (SHR 1.022–15.041). The cohort had a mean age of 69.7 years, with 52.2% male patients. Common comorbidities included hypertension (79.3%), diabetes (31.4%), congestive heart failure (18.8%), and sepsis (35.0%). Compared to Group 1, Group 2 patients exhibited significantly higher heart and respiratory rates, as well as elevated white blood cell counts and urea nitrogen levels, indicating greater physiological stress. Additionally, Group 2 had higher rates of liver disease and hyperlipidemia, suggesting a greater burden of underlying health conditions. Notably, in-hospital procedures differed significantly between the two groups: Group 2 patients had higher rates of mechanical ventilation initiated on the first ICU-day (27.4% vs. 16.4%, *P* < 0.001), craniotomy (9.9% vs. 4.3%, *P* < 0.001), and ventricular drainage (2.8% vs. 1.1%, *P* = 0.002), indicating more intensive treatment. Baseline characteristics were consistent before and after imputation, indicating that the imputation process did not influence overall group comparisons or key variables, thus supporting the robustness of the dataset for further analysis.

**Table 1:** Characteristics and outcomes of participants by SHR category, before and after imputation.

**Characteristic**	**Before Imputation**				**After Imputation**			
	**Total (*N =* 2,423)**	**Group 1 (*N =* 1,211)**	**Group 2 (*N =* 1,212)**	***P*-value**	**Total (*N =* 2,423)**	**Group 1 (*N =* 1,211)**	**Group 1 (*N =* 1,212)**	***P*-value**
**Demographic**								
Sex (Male)	1,265 (52.2)	633 (52.3)	632 (52.1)	0.951	1,265 (52.2)	633 (52.3)	632 (52.1)	0.951
Age	69.7 ± 15.4	70.6 ± 15.2	68.8 ± 15.5	0.004	69.7 ± 15.4	70.6 ± 15.2	68.8 ± 15.5	0.004
Weight^*^	80.2 ± 30.4	79.5 ± 36.9	80.9 ± 22.1	0.244	80.2 ± 30.4	79.4 ± 36.9	81.0 ± 22.1	0.217
**Vital signs**								
HR (bpm)^*^	80.0 ± 14.7	77.6 ± 13.7	82.5 ± 15.2	<0.001	80.0 ± 14.7	77.6 ± 13.7	82.5 ± 15.2	<0.001
MBP^*^	88.2 ± 11.4	89.4 ± 11.4	87.1 ± 11.3	<0.001	88.2 ± 11.4	89.4 ± 11.4	87.1 ± 11.3	<0.001
RR (bpm)^*^	18.9 ± 3.0	18.5 ± 2.8	19.2 ± 3.2	<0.001	18.9 ± 3.0	18.5 ± 2.8	19.2 ± 3.2	<0.001
Temperature^*^	37.0 ± 0.4	37.0 ± 0.4	37.0 ± 0.4	0.001	37.0 ± 0.4	37.0 ± 0.4	37.0 ± 0.4	0.001
SpO_2_ (%)^*^	97.0 ± 1.8	96.8 ± 1.8	97.2 ± 1.8	<0.001	97.0 ± 1.8	96.8 ± 1.8	97.2 ± 1.8	<0.001
**Laboratory tests**								
Hemoglobin^*^	11.9 ± 2.2	12.1 ± 2.0	11.7 ± 2.3	<0.001	11.9 ± 2.2	12.1 ± 2.0	11.7 ± 2.3	<0.001
Platelets^*^	208.6 ± 77.3	215.6 ± 74.4	201.6 ± 79.5	<0.001	208.6 ± 77.3	215.6 ± 74.4	201.7 ± 79.4	<0.001
RBC^*^	3.9 ± 1.0	4.0 ± 1.0	3.8 ± 0.9	<0.001	3.9 ± 1.0	4.0 ± 1.0	3.8 ± 0.9	<0.001
WBC^*^	12.0 ± 7.0	10.7 ± 7.7	13.3 ± 6.0	<0.001	12.0 ± 7.0	10.7 ± 7.7	13.3 ± 6.0	<0.001
Urea nitrogen^*^	21.4 ± 14.4	20.1 ± 12.3	22.7 ± 16.2	<0.001	21.4 ± 14.4	20.1 ± 12.3	22.7 ± 16.2	<0.001
Creatinine^#*^	0.9 (0.8, 1.2)	0.9 (0.8, 1.2)	1.0 (0.8, 1.2)	0.093	0.9 (0.8, 1.2)	0.9 (0.8, 1.2)	1.0 (0.8, 1.2)	0.103
Sodium^*^	138.1 ± 4.4	138.5 ± 4.0	137.7 ± 4.7	<0.001	138.1 ± 4.4	138.5 ± 4.0	137.7 ± 4.7	<0.001
Potassium^*^	3.8 ± 0.5	3.9 ± 0.5	3.8 ± 0.5	0.009	3.8 ± 0.5	3.9 ± 0.5	3.8 ± 0.5	0.005
AST^#*^	19.0 (14.0, 30.0)	18.0 (13.0, 28.0)	21.0 (14.0, 32.0)	<0.001	19.0 (14.0, 29.0)	18.0 (13.0, 28.0)	20.5 (14.0, 32.0)	<0.001
Glucose	142.5 ± 71.1	112.8 ± 33.0	172.1 ± 85.2	<0.001	142.5 ± 71.1	112.8 ± 33.0	172.1 ± 85.2	<0.001
HbA1c	6.2 ± 1.5	6.3 ± 1.5	6.1 ± 1.4	<0.001	6.2 ± 1.5	6.3 ± 1.5	6.1 ± 1.4	<0.001
**Medical history**								
Smoke	756 (31.2)	393 (32.5)	363 (30)	0.184	756 (31.2)	393 (32.5)	363 (30)	0.184
**Organ dysfunction**								
Dementia	169 (7.0)	88 (7.3)	81 (6.7)	0.573	169 (7.0)	88 (7.3)	81 (6.7)	0.573
CBD	2,316 (95.6)	1,174 (96.9)	1,142 (94.2)	0.001	2,316 (95.6)	1,174 (96.9)	1,142 (94.2)	0.001
Cancer	152 (6.3)	67 (5.5)	85 (7)	0.133	152 (6.3)	67 (5.5)	85 (7)	0.133
Rheumatic	45 (1.9)	19 (1.6)	26 (2.1)	0.293	45 (1.9)	19 (1.6)	26 (2.1)	0.293
Liver disease	104 (4.3)	32 (2.6)	72 (5.9)	<0.001	104 (4.3)	32 (2.6)	72 (5.9)	<0.001
Hyperlipidemia	1,139 (47.0)	609 (50.3)	530 (43.7)	0.001	1,139 (47.0)	609 (50.3)	530 (43.7)	0.001
Diabetes	762 (31.4)	359 (29.6)	403 (33.3)	0.056	762 (31.4)	359 (29.6)	403 (33.3)	0.056
HBP	1,921 (79.3)	955 (78.9)	966 (79.7)	0.609	1,921 (79.3)	955 (78.9)	966 (79.7)	0.609
MI	293 (12.1)	140 (11.6)	153 (12.6)	0.422	293 (12.1)	140 (11.6)	153 (12.6)	0.422
CHF	455 (18.8)	227 (18.7)	228 (18.8)	0.966	455 (18.8)	227 (18.7)	228 (18.8)	0.966
Sepsis3	849 (35.0)	329 (27.2)	520 (42.9)	<0.001	849 (35.0)	329 (27.2)	520 (42.9)	<0.001
**Score**								
CCI	6.1 ± 2.7	6.2 ± 2.7	6.1 ± 2.8	0.523	6.1 ± 2.7	6.2 ± 2.7	6.1 ± 2.8	0.523
GCS	11.1 ± 3.5	11.8 ± 3.2	10.5 ± 3.7	<0.001	11.1 ± 3.5	11.8 ± 3.2	10.5 ± 3.7	<0.001
SAPS II	32.5 ± 11.3	30.9 ± 10.5	34.1 ± 11.9	<0.001	32.5 ± 11.3	30.9 ± 10.5	34.1 ± 11.9	<0.001
**In-hospital procedures**								
Vent1day	530 (21.9)	198 (16.4)	332 (27.4)	<0.001	530 (21.9)	198 (16.4)	332 (27.4)	<0.001
Craniotomy	172 (7.1)	52 (4.3)	120 (9.9)	<0.001	172 (7.1)	52 (4.3)	120 (9.9)	<0.001
Pe	357 (14.7)	162 (13.4)	195 (16.1)	0.06	357 (14.7)	162 (13.4)	195 (16.1)	0.06
Vd	47 (1.9)	13 (1.1)	34 (2.8)	0.002	47 (1.9)	13 (1.1)	34 (2.8)	0.002
Diuretic	818 (33.8)	346 (28.6)	472 (38.9)	<0.001	818 (33.8)	346 (28.6)	472 (38.9)	<0.001
β_blocker	1653 (68.2)	759 (62.7)	894 (73.8)	<0.001	1653 (68.2)	759 (62.7)	894 (73.8)	<0.001

SHR, Stress Hyperglycemia Ratio; ABI, acute brain injury; ICU, intensive care unit; HR, heart rate (beats per minute, bpm); MBP, mean blood pressure (mmHg); RR, respiratory rate (breaths per minute, bpm); SpO_2_, oxygen saturation (%); WBC, white blood cell count (10^9^/L); RBC, red blood cell count (10^1^2/L); Hemoglobin (g/dL); Platelets (10^9^/L); Creatinine (mg/dL); Urea nitrogen (mg/dL); Sodium (mmol/L); Potassium (mmol/L); AST, aspartate aminotransferase (U/L); MI, myocardial infarction; CHF, congestive heart failure; CBD, Cerebrovascular disease; HBP, hypertension; Sepsis3, Sepsis clinical criteria from The Third International Consensus Definitions for Sepsis and Septic Shock; CCI, Charlson comorbidity index; SAPS II, simplified acute physiological score II; GCS, Glasgow coma scale; Vent1day, ventilation on the first day of ICU admission; Pe, percutaneous cerebral arterial embolization; Vd, ventricular drainage.

Values are presented as mean ± standard deviation (SD), median [interquartile range, IQR], or number (%). ^*^Variables with <5% missing data include Heart rate, MBP, Respiratory rate, SpO_2_, Temperature, Weight, WBC, Hemoglobin, Platelets, Urea nitrogen, Creatinine, Potassium and Sodium. Variables with 20–30% missing data include AST. ^#^Due to non-normal distribution, Creatinine and AST values are presented as median [IQR]. Group comparisons were assessed with appropriate statistical tests, with p-values <0.05 indicating statistical significance.

### Clinical outcomes

**Figure 1:**
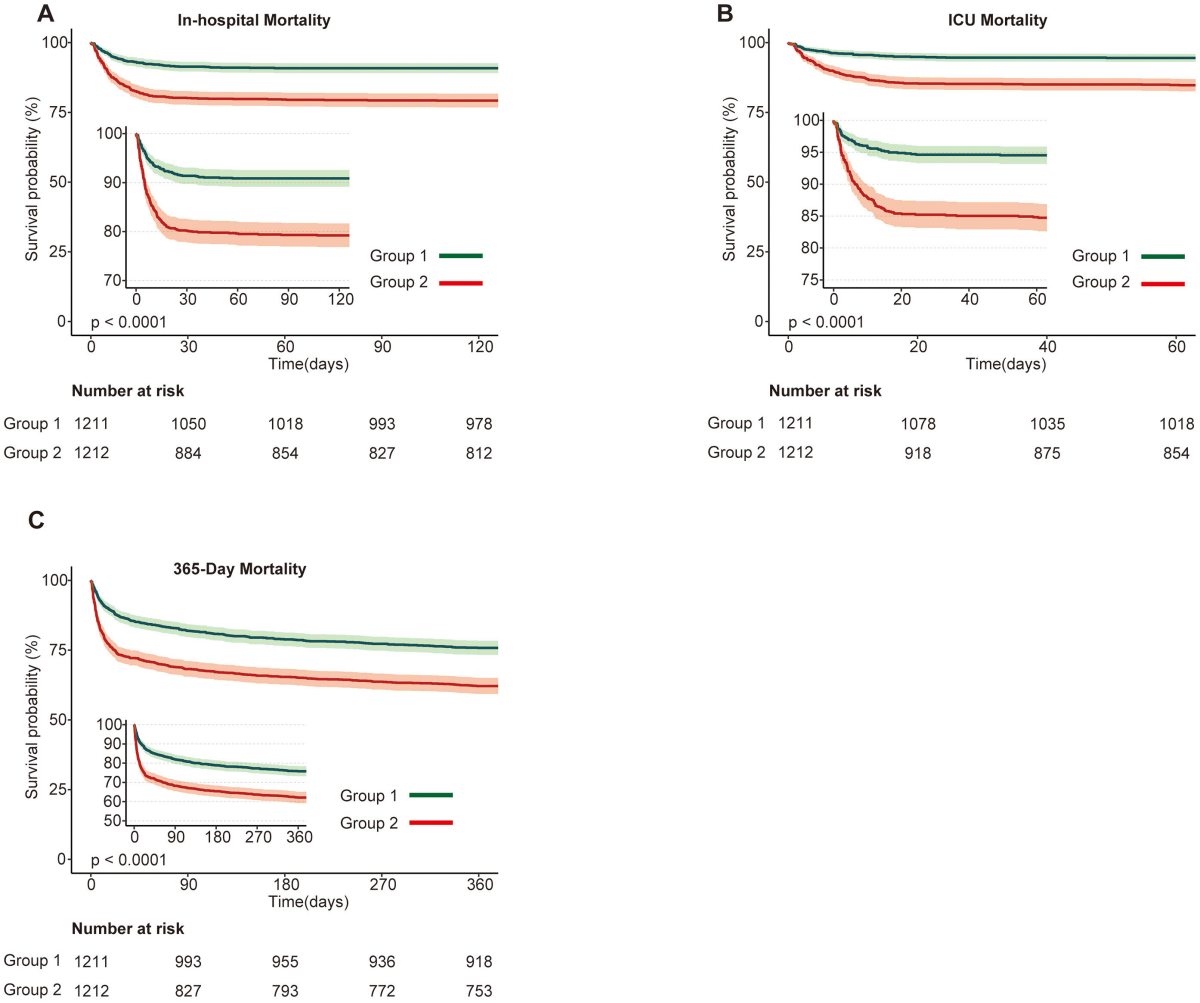
Kaplan-Meier survival curves for primary mortality outcomes stratified by SHR groups in patients with acute brain injury. **(A)** In-hospital mortality; **(B)** ICU mortality; **(C)** 365-day mortality. Survival probabilities are compared between Group 1 (green) and Group 2 (red), with Group 1 showing higher survival probabilities across all outcomes. The number at risk at each time point is displayed below the plots. Statistically significant differences in survival are observed between the groups (*p* < 0.0001).

After baseline assessment, associations between SHR and various mortality outcomes were analyzed in the cohort of 2,423 ABI patients. As shown in Supplementary Figure 2, the proportional hazards assumption was tested and confirmed using log-log survival plots and Schoenfeld residuals. The test for SHR yielded a chi-square of 0.587 (*p* = 0.444), validating the use of Cox regression models to explore the relationship between SHR and mortality outcomes. Kaplan-Meier survival curves for short- and long-term mortality outcomes (in-hospital, ICU, 30, 60, 90, and 365-day) demonstrated significantly lower survival probabilities in the higher SHR group (Group 2) compared to the lower SHR group (Group 1). Log-rank tests confirmed significant differences across all outcomes (*p* < 0.001; Figure 1, Supplementary Figure 3).

Cox regression analysis was performed, incorporating variables with *p* < 0.1 from univariate analysis and clinically significant covariates (Supplementary Table 2), with multiple imputation used to address missing data. As shown in Table 2 and Supplementary Table 3, higher SHR levels were significantly associated with increased risks for both short- and long-term mortality outcomes. When analyzed as a continuous variable, SHR consistently demonstrated a significant positive association with in-hospital (HR: 1.18, 95% CI: 1.06–1.32, *P* = 0.003), ICU (HR: 1.16, 95% CI: 1.02–1.32, *P* = 0.029), and 365-day mortality (HR: 1.14, 95% CI: 1.05–1.23, *P* = 0.002) in fully adjusted models (Model 3). When dichotomized, higher SHR (Group 2 vs. Group 1) was associated with increased mortality risks for in-hospital (HR: 1.44, 95% CI: 1.13–1.83, *P* = 0.003), ICU (HR: 1.50, 95% CI: 1.10–2.04, *P* = 0.009), and 365-day mortality (HR: 1.33, 95% CI: 1.14–1.56, P <0.001).

**Table 2:** Association of SHR with short-term and long-term mortality risk.

**Categories**	**Model1**		**Model2**		**Model3**	
	**HR (95%CI)**	***P*-value**	**HR (95%CI)**	***P*-value**	**HR (95%CI)**	***P*-value**
**In-hospital mortality**						
SHR (continuous variable)	1.25 (1.17–1.34)	<0.001	1.23 (1.11–1.36)	<0.001	1.18 (1.06–1.32)	0.003
**SHR (dichotomized)**						
Group1	Ref	Ref	Ref	Ref	Ref	Ref
Group2	1.79 (1.43–2.24)	<0.001	1.58 (1.25–2.01)	<0.001	1.44 (1.13–1.83)	0.003
**ICU mortality**						
SHR (continuous variable)	1.21 (1.11–1.31)	<0.001	1.24 (1.1–1.39)	<0.001	1.16 (1.02–1.32)	0.029
**SHR (dichotomized)**						
Group1	Ref	Ref	Ref	Ref	Ref	Ref
Group2	1.86 (1.4–2.48)	<0.001	1.65 (1.22–2.22)	0.001	1.5 (1.1–2.04)	0.009
**365-day mortality**						
SHR (continuous variable)	1.31 (1.24–1.38)	<0.001	1.19 (1.11–1.29)	<0.001	1.14 (1.05–1.23)	0.002
**SHR (dichotomized)**						
Group1	Ref	Ref	Ref	Ref	Ref	Ref
Group2	1.77 (1.53–2.05)	<0.001	1.45 (1.24–1.7)	<0.001	1.33 (1.14–1.56)	<0.001
**30-day mortality**						
SHR (continuous variable)	1.31 (1.24–1.38)	<0.001	1.22 (1.13–1.33)	<0.001	1.16 (1.07–1.27)	0.001
**SHR (dichotomized)**						
Group1	Ref	Ref	Ref	Ref	Ref	Ref
Group2	2.24 (1.85–2.7)	<0.001	1.79 (1.46–2.18)	<0.001	1.61 (1.31–1.97)	<0.001
**60-day mortality**						
SHR (continuous variable)	1.31 (1.24–1.38)	<0.001	1.22 (1.13–1.32)	<0.001	1.16 (1.06–1.26)	0.001
**SHR (dichotomized)**						
Group1	Ref	Ref	Ref	Ref	Ref	Ref
Group2	2.05 (1.72–2.45)	<0.001	1.66 (1.37–1.99)	<0.001	1.49 (1.23–1.8)	<0.001
**90-day mortality**						
SHR (continuous variable)	1.31 (1.24–1.38)	<0.001	1.21 (1.11–1.3)	<0.001	1.15 (1.05–1.25)	0.002
**SHR (dichotomized)**						
Group1	Ref	Ref	Ref	Ref	Ref	Ref
Group2	1.97 (1.67–2.32)	<0.001	1.58 (1.32–1.88)	<0.001	1.43 (1.2–1.71)	<0.001

SHR, Stress hyperglycemia ratio; HR, hazard ratio; CI, confidence interval; ABI, acute brain injury; ICU, intensive care unit; MBP, mean blood pressure; SpO_2_, oxygen saturation; RBC, red blood cell; WBC, white blood cell; BUN, blood urea nitrogen; SAPS II, simplified acute physiological score II; GCS, Glasgow coma scale. Model Descriptions: Model 1, unadjusted; Model 2, adjusted for admission age, weight, heart rate, MBP, respiratory rate, SpO_2_, hemoglobin, platelets, RBC, WBC, BUN, creatinine, sodium, liver disease, hypertension, diabetes, and Sepsis3; Model 3, adjusted for Model 2 covariates plus Charlson comorbidity index, GCS, SAPS II, ventilation on the first ICU day, craniotomy, and diuretic use in the ICU.

For other secondary endpoints, including 30, 60, and 90-day mortality, similar patterns were observed, with higher SHR consistently associated with increased risks. For example, as a continuous variable, SHR demonstrated significant associations with 30-day mortality (HR: 1.16, 95% CI: 1.07–1.27, *P* = 0.001), 60-day mortality (HR: 1.16, 95% CI: 1.06–1.26, *P* = 0.001), and 90-day mortality (HR: 1.15, 95% CI: 1.05–1.25, *P* = 0.002). Dichotomized SHR analysis similarly showed elevated risks across these endpoints. Stepwise regression analysis, as part of a sensitivity analysis, identified relevant predictors of mortality outcomes and adjusted for them in the final models, as presented in Supplementary Tables 4, 5. These consistent findings across multiple outcomes underscore the robustness of the observed association between SHR and mortality, further supporting the reliability of the study conclusions.

**Figure 2:**
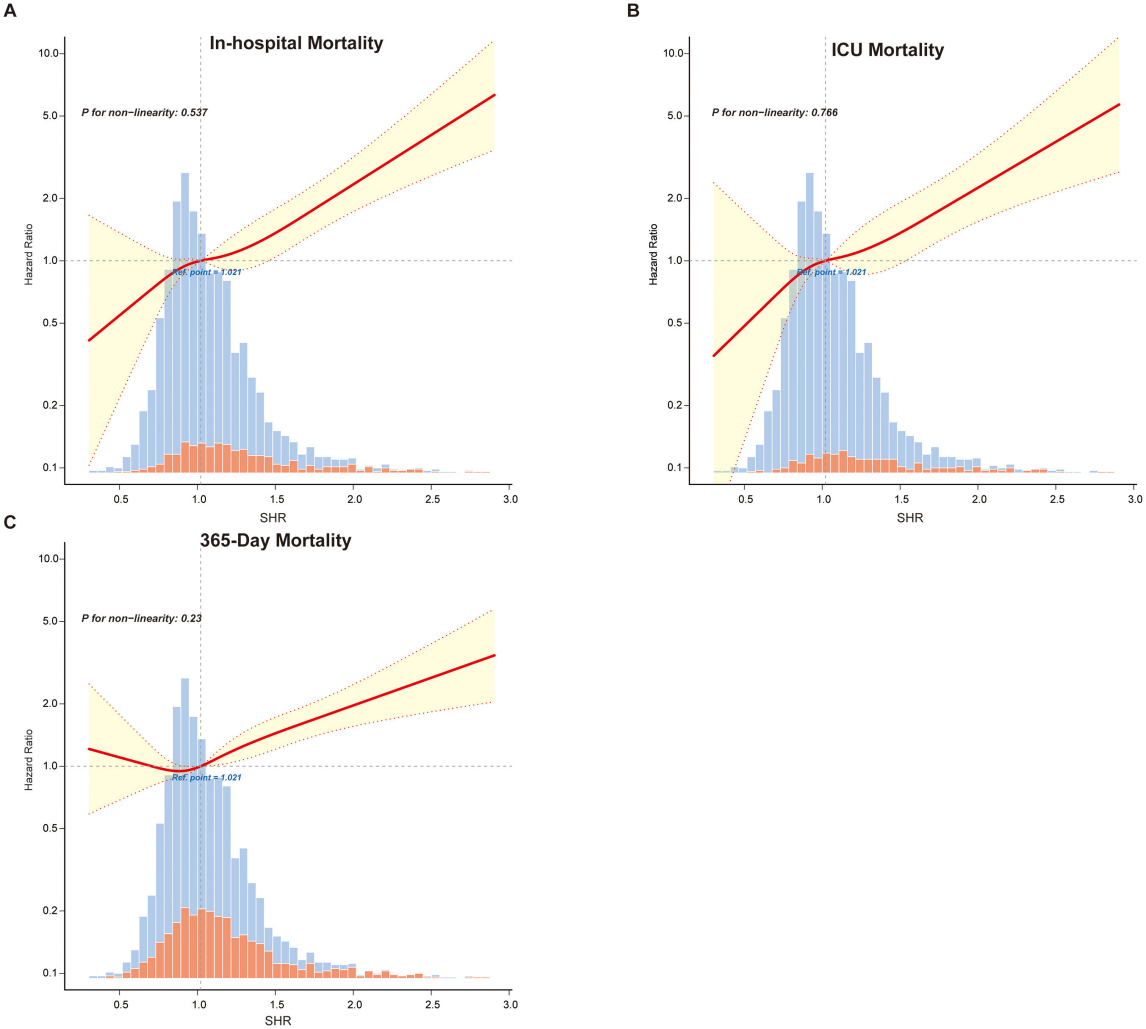
Restricted cubic spline curves illustrating the linear relationship between SHR and primary outcomes: **(A)** in-hospital mortality, **(B)** ICU mortality and **(C)** 365-Day mortality. Vertical dashed lines indicate reference points, the red line represents fully adjusted hazard ratios, and the shaded yellow area shows the 95% confidence interval.

Restricted cubic spline analyses (Figure 2 and Supplementary Figure 4) confirmed a linear relationship between SHR and mortality outcomes, reinforcing SHR's predictive value. The fitted hazard ratios (HR) with 95% confidence intervals consistently indicated higher mortality risks with increasing SHR values, with no evidence of non-linearity (*P* for non-linearity > 0.05 across all mortality outcomes).

### Subgroup analysis

**Figure 3:**
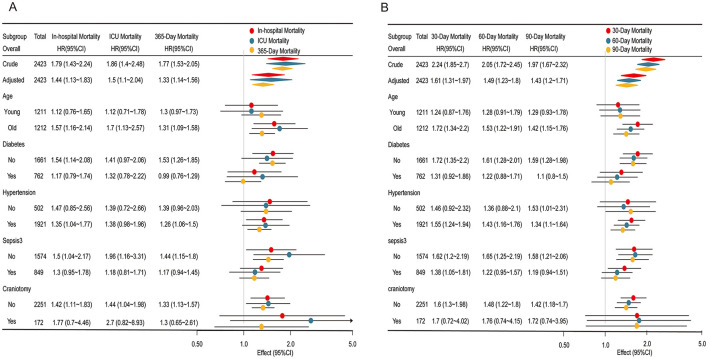
Subgroup analyses of the association between the stress hyperglycemia ratio (SHR) and mortality outcomes. **(A)** Association of SHR with in-hospital mortality, ICU mortality, and 365-day mortality. **(B)** Association of SHR with 30, 60, and 90-day mortality. Each subgroup analysis displays the Hazard ratios (HR) and 95% confidence intervals (CI) for different mortality outcomes, stratified by age, diabetes status, hypertension, sepsis, and craniotomy status. Colored markers represent mortality types: red for in-hospital/30-day mortality, blue for ICU/60-day mortality, and yellow for 365-day/90-day mortality. Significant associations are highlighted, with effect estimates plotted on a logarithmic scale for clarity.

Subgroup analyses supported the primary findings, as part of our sensitivity analysis, further validating the relationship between SHR and mortality across various patient subgroups (Figure 3). Stratifications by age, diabetes status, hypertension, sepsis, and craniotomy provided comprehensive insights into SHR's predictive capacity in different clinical settings. Across all subgroups, higher SHR values were consistently linked to increased risks of in-hospital, ICU, 30, 60, 90, and 365-day mortality.

For in-hospital, ICU, and 365-day mortality (Figure 3A), the association between elevated SHR and higher mortality risk was most pronounced in older and non-diabetic patients, with odds ratios slightly higher than those observed in younger or diabetic patients. Interaction *p-*values exceeded 0.05, reinforcing the stability of the linear relationship between SHR and mortality across these subgroups. Similarly, for 30, 60, and 90-day mortality (Figure 3B), the association remained consistent across all subgroups, further confirming SHR's robustness as a predictor of mortality across different patient characteristics.

### SHR and its combined use with GCS and Vent1day in predicting mortality in ABI patients

**Figure 4:**
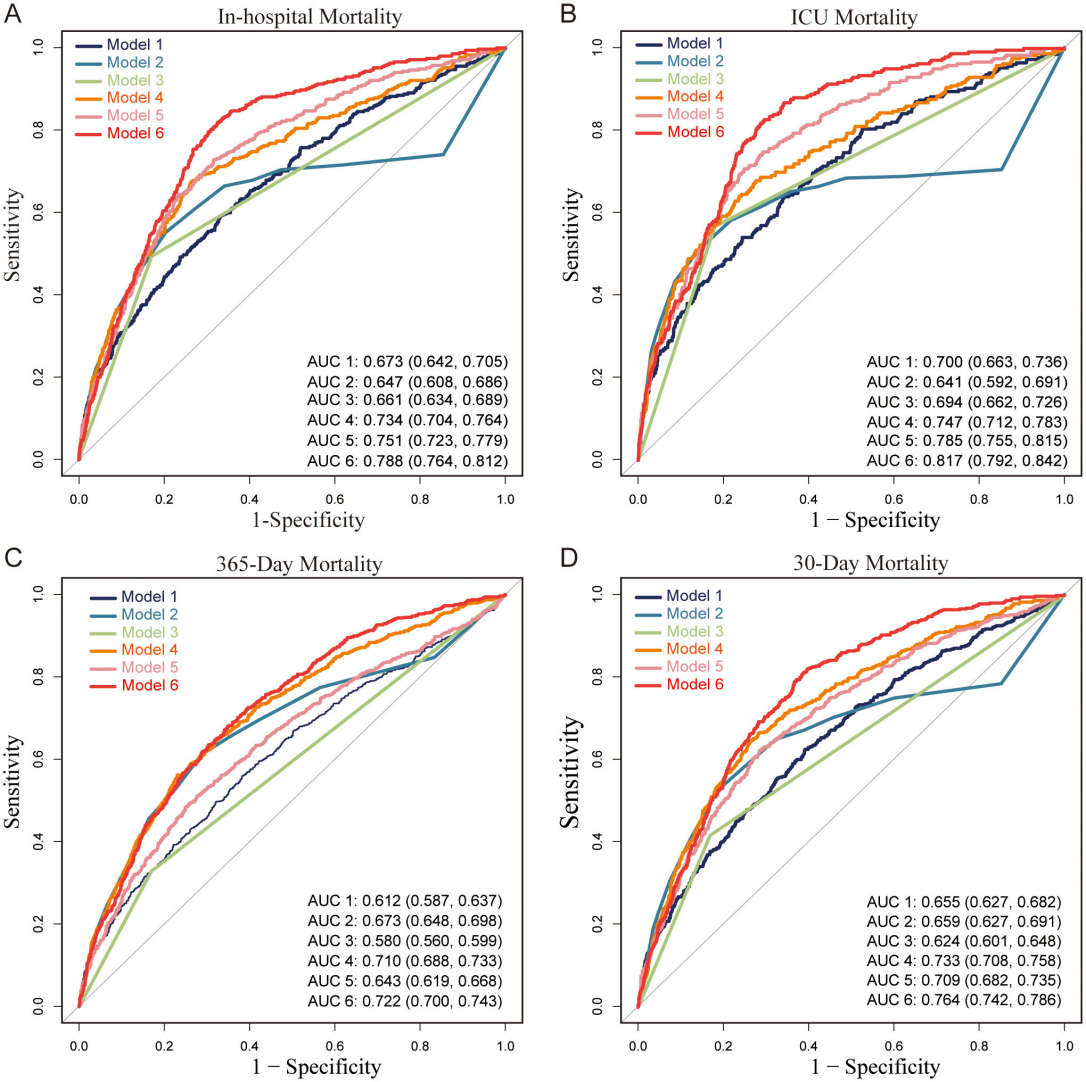
Receiver Operating Characteristic (ROC) curves for predictive models of mortality in patients with acute brain injury (ABI). **(A)** In-hospital mortality, **(B)** ICU mortality, **(C)** 365-day mortality, and **(D)** 30-day mortality. Abbreviations: ABI, acute brain injury; ICU, intensive care unit; AUC, area under the curve; SHR, stress hyperglycemia ratio; GCS, Glasgow Coma Scale; Vent1day, ventilation initiated on the first ICU admission. Model Descriptions: Model 1: SHR only; Model 2: GCS only; Model 3: Vent1day only; Model 4: SHR and GCS combined; Model 5: SHR and Vent1day combined; Model 6: SHR, GCS, and Vent1day combined. Notes: Each ROC curve illustrates the sensitivity and specificity of six predictive models for different mortality outcomes in ABI patients. The AUC value indicates each model's discriminatory ability, with higher values reflecting better predictive performance. Model 6 consistently achieves the highest AUC across all mortality outcomes, indicating the strongest predictive accuracy.

As shown in Table 3, SHR demonstrated strong independent predictive value across all mortality outcomes. GCS and Vent1day were selected based on their clinical significance and their established role in predicting mortality, especially in critically ill neurosurgical patients (10, 11). Model 1 (SHR alone) achieved high AUC values, surpassing both the GCS-based (Model 2) and Vent1day-based (Model 3) models. For in-hospital mortality, Model 1 achieved an AUC of 0.673 (95% CI: 0.642–0.705), outperforming Model 2 (AUC: 0.647) and Model 3 (AUC: 0.661). Combining SHR with GCS and Vent1day further enhanced predictive accuracy, as evidenced by the highest AUC values in Model 6 across both short- and long-term mortality outcomes. For instance, Model 6 achieved an AUC of 0.817 (95% CI: 0.792–0.842) for ICU mortality and 0.788 (95% CI: 0.764–0.812) for in-hospital mortality, underscoring the enhanced predictive power of integrating multiple markers. The ROC curves in Figure 4 and Supplementary Figure 5 visually confirmed this pattern, with Model 6 consistently exhibiting superior discriminatory power across various mortality endpoints in ABI patients. To clarify, we have revised the original manuscript to include the following statement: Model 1 (SHR alone) shows moderate performance with accuracy of 0.657, specificity of 0.669, and sensitivity of 0.590. When SHR is combined with GCS and Vent1day in Model 6, accuracy increases to 0.695, and specificity rises to 0.819, with sensitivity also improving to 0.819. These improvements demonstrate that combining these variables enhances predictive accuracy for mortality outcomes. All key performance metrics—accuracy, specificity, and sensitivity—are shown for each model across different mortality outcomes in Table 3. As part of a sensitivity analysis, LASSO regression was performed to further validate the variable selection, ensuring that the identified variables, including SHR, GCS, and Vent1day, were robust and critical predictors for mortality outcomes (shown in Supplementary Figure 6). Additionally, Time-dependent AUC analysis for predicting in-hospital mortality was performed as a sensitivity analysis (Supplementary Figure 7) to further validate the incremental predictive value of SHR.

**Table 3:** Performance of SHR, GCS, and Vent1day models in predicting short-term and long-term mortality.

**Models**	**Specificity**	**Sensitivity**	**Accuracy**	**Precision**	**Recall**	**AUC**
**In-hospital mortality**						
Model 1	0.669	0.590	0.657	0.234	0.590	0.673 (0.642–0.705)
Model 2	0.797	0.551	0.761	0.317	0.551	0.647 (0.608–0.686)
Model 3	0.828	0.494	0.780	0.330	0.494	0.661 (0.634–0.689)
Model 4	0.736	0.675	0.727	0.304	0.675	0.734 (0.704–0.764)
Model 5	0.683	0.729	0.690	0.282	0.729	0.751 (0.723–0.779)
Model 6	0.674	0.819	0.695	0.301	0.819	0.788 (0.764–0.812)
**ICU mortality**						
Model 1	0.657	0.638	0.655	0.172	0.638	0.700 (0.663–0.736)
Model 2	0.856	0.510	0.822	0.284	0.510	0.641 (0.592–0.691)
Model 3	0.820	0.568	0.795	0.260	0.568	0.694 (0.662–0.726)
Model 4	0.762	0.642	0.750	0.232	0.642	0.747 (0.712–0.783)
Model 5	0.751	0.708	0.747	0.241	0.708	0.785 (0.755–0.815)
Model 6	0.713	0.819	0.724	0.241	0.819	0.817 (0.792–0.842)
**365-day mortality**						
Model 1	0.616	0.560	0.599	0.396	0.560	0.612 (0.587–0.637)
Model 2	0.712	0.612	0.681	0.489	0.612	0.673 (0.648–0.698)
Model 3	0.831	0.329	0.675	0.466	0.329	0.580 (0.560–0.599)
Model 4	0.770	0.563	0.705	0.524	0.563	0.710 (0.688–0.733)
Model 5	0.724	0.512	0.658	0.455	0.512	0.643 (0.619–0.668)
Model 6	0.697	0.636	0.678	0.485	0.636	0.722 (0.700–0.743)
**30-day mortality**						
Model 1	0.609	0.624	0.612	0.287	0.624	0.655 (0.627–0.682)
Model 2	0.814	0.524	0.756	0.416	0.524	0.659 (0.627–0.691)
Model 3	0.831	0.417	0.748	0.385	0.417	0.624 (0.601–0.648)
Model 4	0.717	0.667	0.707	0.373	0.667	0.733 (0.708–0.758)
Model 5	0.715	0.624	0.697	0.356	0.624	0.709 (0.682–0.735)
Model 6	0.613	0.812	0.653	0.347	0.812	0.764 (0.742–0.786)
**60-day mortality**						
Model 1	0.610	0.601	0.608	0.312	0.601	0.641 (0.614–0.668)
Model 2	0.689	0.652	0.681	0.382	0.652	0.669 (0.639–0.699)
Model 3	0.832	0.392	0.732	0.408	0.392	0.612 (0.590–0.634)
Model 4	0.740	0.639	0.717	0.420	0.639	0.730 (0.706–0.754)
Model 5	0.713	0.597	0.686	0.380	0.597	0.690 (0.664–0.716)
Model 6	0.696	0.701	0.697	0.404	0.701	0.754 (0.732–0.776)
**90-day mortality**						
Model 1	0.676	0.529	0.639	0.351	0.529	0.633 (0.607–0.660)
Model 2	0.697	0.647	0.685	0.415	0.647	0.675 (0.647–0.703)
Model 3	0.834	0.377	0.720	0.428	0.377	0.605 (0.584–0.626)
Model 4	0.748	0.620	0.717	0.450	0.620	0.727 (0.703–0.750)
Model 5	0.719	0.569	0.681	0.401	0.569	0.679 (0.653–0.704)
Model 6	0.667	0.723	0.681	0.418	0.723	0.748 (0.727–0.770)

ABI, acute brain injury; ICU, intensive care unit; HR, hazard ratio; CI, confidence interval; AUC, area under the curve.

Model descriptions: Model 1: based on SHR; Model 2: based on Glasgow Coma Scale (GCS); Model 3: based on ventilation on the first ICU day (Vent1day); Model 4: combined SHR and GCS; Model 5: combined SHR and Vent1day; Model 6: combined SHR, GCS, and Vent1day.

Supplementary Figure 8 presents the *E-*values from Table 4, demonstrating SHR's resilience to unmeasured confounding across all mortality outcomes. The *E-*value represents the minimum strength of association that an unmeasured confounder would need to have with both SHR and the mortality outcome to fully explain the observed relationship. For ICU mortality, SHR had an *E-*value of 2.37 (lower CI: 1.43), indicating that even a moderate unmeasured confounder would need a strong association with both SHR and ICU mortality to nullify the observed association. Similarly, for in-hospital mortality, the *E-*value was 2.24 (lower CI: 1.51). Additional results include 30-day mortality (E-value: 2.60, lower CI: 1.95), 60-day mortality (E-value: 2.34, lower CI: 1.76), 90-day mortality (E-value: 2.21, lower CI: 1.69), and 365-day mortality (E-value: 1.99, lower CI: 1.54), further reinforcing SHR's predictive strength across both short-term and long-term outcomes. These findings highlight that the association between SHR and mortality is robust and not likely to be significantly impacted by unmeasured confounding.

**Table 4:** E-values for SHR as a predictor of short-term and long-term mortality.

**Outcomes**	**In-hospital mortality**	**ICU mortality**	**365-day mortality**	**30-day mortality**	**60-day mortality**	**90-day mortality**
E-value (Lower CI)	2.24 (1.51)	2.37 (1.43)	1.99 (1.54)	2.60 (1.95)	2.34 (1.76)	2.21 (1.69)

## Discussion

In this study, we demonstrated that the SHR is a reliable, independent predictor of mortality at various time points: in-hospital, ICU, 30, 60, 90, and 365-day outcomes in patients with ABI. The linear association between SHR and mortality, consistent across various patient subgroups, highlights SHR's stability and its potential utility as a robust biomarker for mortality risk stratification with ABI. These findings support and extend previous studies, further validating SHR's role in reflecting acute metabolic stress in critically ill brain-injured patients. The consistency of SHR's association with mortality across subgroups, combined with its linear relationship to mortality, indicates its reliable predictive capacity, distinguishing it from other glycemic metrics.

Our results build on the findings of Rau et al. and Pan et al., who associated stress hyperglycemia with increased mortality in traumatic brain injury and ischemic stroke, highlighting SHR's superior predictive ability compared to conventional glucose metrics (8, 12). In contrast to these studies, our analysis expands the prognostic utility of SHR by covering a diverse ABI cohort that includes both stroke and trauma cases. Similar ABI cohorts have been utilized in high-impact studies, which further underscores the relevance of our cohort (1). Comparative studies have revealed the diverse relationships between SHR and mortality in different critical populations (13, 14). For example, in acute myocardial infarction (AMI) patients, a J-shaped association was observed between SHR and all-cause mortality, with both high and low SHR values associated with elevated risk, particularly in non-diabetic individuals (7). Likewise, Zhang et al. observed a clearly non-linear, potentially J-shaped, association in patients with acute coronary syndrome and triple-vessel disease, with elevated SHR posing a significant cardiovascular mortality risk, especially among diabetic patients (15). In contrast to this, Le Li's study on sepsis reported a U-shaped association between SHR and 1-year mortality, with an SHR of 0.99 as the inflection point; both high and low SHR values were associated with increased mortality risk, improving the predictive accuracy of conventional severity scores (16). In a similar vein, Climent et al. reported that higher acute-to-chronic glycemic ratio (ACR) values were associated with worse outcomes in ischemic stroke patients, indicating a steady increase in risk without the non-linear patterns observed in AMI and coronary disease (17).

Our study highlights a stable linear association between the SHR and mortality across multiple time points in ABI patients, with no significant interaction effects across subgroups. This finding supports SHR as a consistent and independent predictor of mortality risk in ABI. In contrast, our previous research demonstrated a U-shaped association between GV and mortality in non-diabetic patients, with both high and low GV levels elevating mortality risk, emphasizing the need for population-specific glycemic assessment. This underscores the importance of population-specific assessment in glycemic monitoring, as different glycemic metrics may have varying implications depending on patient characteristics (8, 18, 19). Consistent with our findings, several studies have repeatedly demonstrated SHR's prognostic value across diverse critical conditions and populations. For example, Ding et al. found that SHR is significantly associated with all-cause and cardiovascular mortality in diabetic and prediabetic patients, reinforcing its predictive value in glucose-sensitive populations (20). Additionally, research on coronary artery disease patients showed that combined assessment of SHR and GV provided superior prognostic accuracy, with non-diabetic individuals experiencing the greatest risk of in-hospital and 1-year mortality when both SHR and GV were elevated (6). Furthermore, a study in acute myocardial infarction (AMI) patients demonstrated that elevated fasting SHR strongly correlated with in-hospital mortality in both diabetic and non-diabetic groups, highlighting SHR's value as a robust risk stratification tool across glucose metabolism statuses (3).

ABI is associated with high incidence and poor prognosis, driving significant research efforts to develop more effective prognostic models (21, 22). The Glasgow Coma Scale (GCS) and early mechanical ventilation are among the most accessible ICU indicators for ABI outcomes (23, 24). Previous studies have demonstrated that combining multiple monitoring parameters can enhance predictive accuracy; however, these models often rely on complex metrics, which may limit their clinical applicability (25–28). By contrast, our model, which combines the SHR with GCS and Vent1day, achieves strong predictive performance using readily available indicators. This approach provides a practical and feasible tool for routine use in ABI prognosis, supporting its broader adoption in critical care settings (29, 30).

The observed linear relationship between SHR and mortality in ABI patients suggests a direct impact of stress-induced hyperglycemia on adverse outcomes, potentially mediated through neuroendocrine activation (31), oxidative stress (32), and inflammation (22, 33). Glucose is critical for brain function (34), supporting ATP production and neurotransmitter synthesis. However, stress disrupts normal metabolism, increasing reliance on glycolysis and the pentose phosphate pathway, which may intensify oxidative damage and neuroinflammation (2, 35). The hypothalamus-sympathetic-liver (HSL) axis rapidly mobilizes glucose in response to stress, independent of adrenal activity, providing an immediate energy supply. However, potentially exacerbating neuroinflammation and endothelial dysfunction when prolonged (36). Additionally, glucose-sensing alterations to non-diabetic patients may modify the threshold for detecting glycemic extremes, amplifying the impact of hyperglycemia on ABI outcomes (37). Together, these mechanisms underscore SHR's prognostic value and highlight the importance of tailored glycemic management in ABI.

## Strengths and limitations

Although our study provides compelling evidence for the prognostic utility of SHR in ABI, several limitations should be acknowledged. The retrospective design and reliance on a single-center database may limit the generalizability of our study's findings (5, 38). Prospective, multi-center studies are essential to validate the predictive capability of SHR and its influence on clinical decision-making in various clinical settings. Furthermore, further exploration of the mechanistic pathways linking SHR to ABI outcomes is warranted, as this could uncover novel therapeutic targets to mitigate hyperglycemia-induced damage in this patient population.

## Conclusion

In conclusion, our study confirms SHR as a reliable and independent predictor of mortality in ABI patients, offering a novel approach to mortality risk stratification that incorporates baseline glycemic status. The linear association between SHR and mortality across multiple time points and subgroups further highlights its potential as a stable biomarker in neurocritical care. Integrating SHR into clinical risk assessment may enable clinicians to better identify high-risk patients early and optimize glycemic management strategies. Prospective studies are needed to validate these findings and investigate SHR-guided interventions aimed at improving patient outcomes in ABI, ultimately enhancing survival and recovery in this vulnerable population.

## Data Availability

The datasets presented in this study can be found in online repositories. The names of the repository/repositories and accession number(s) can be found below: https://physionet.org/content/mimiciv/3.1/.
