# Supplementary material for: The stress hyperglycemia ratio as a predictor of short- and long-term mortality in patients with acute brain injury: a retrospective cohort study
Source: Front Neurol. 2025 Apr 28;16:1552462. doi: 10.3389/fneur.2025.1552462 (PMC12066301; doi:10.3389/fneur.2025.1552462)

# Supplementary Table 1. Variables extracted from the MIMIC-IV database

| **Variable Category** | **Abbreviation** | **Full Name / Definition** |
| --- | --- | --- |
| **Demographic** | Sex | Male = 1, Female = 0 |
|  | Age | Age (years) |
|  | Weight | Weight (kg) |
| **Vital Signs** | HR | Heart rate (beats per minute) |
|  | MBP | Mean blood pressure (mmHg) |
|  | RR | Respiratory rate (breaths per minute) |
|  | Temperature | Temperature (°C) |
|  | SpO₂ | Peripheral oxygen saturation (%) |
| **Laboratory Tests** | Hemoglobin | Hemoglobin (g/dL) |
|  | Platelets | Platelet count (×10^9^/L) |
|  | RBC | Red blood cell count (×10¹²/L) |
|  | WBC | White blood cell count (×109/L) |
|  | BUN | Blood urea nitrogen (mg/dL) |
|  | MBP | Mean blood pressure (mmHg) |
|  | Creatinine | Creatinine (mg/dL) |
|  | Sodium | Sodium (mmol/L) |
|  | Potassium | Potassium (mmol/L) |
|  | AST | Aspartate aminotransferase (U/L) |
|  | Glucose | Glucose (mg/dL) |
|  | HbA1c | Glycated hemoglobin (%) |
| **Medical History and Comorbidities** | Smoke | Smoking history |
|  | Dementia | Dementia |
|  | CBD | Cerebrovascular disease |
|  | Cancer | Cancer |
|  | Rheumatic | Rheumatic disease |
|  | Liver disease | Liver disease |
|  | Hyperlipidemia | Hyperlipidemia |
|  | Diabetes | Diabetes mellitus |
|  | HBP | Hypertension |
|  | MI | Myocardial infarction |
|  | CHF | Congestive heart failure |
|  | Sepsis3 | Sepsis (Third international consensus definition) |
| **Clinical Severity Scores** | CCI | Charlson Comorbidity Index |
|  | GCS | Glasgow Coma Scale |
|  | SAPS II | Simplified Acute Physiology Score II |
| **In-hospital Procedures** | Vent1day | Mechanical ventilation on first ICU admission |
|  | Craniotomy | Craniotomy |
|  | Pe | Percutaneous cerebral embolization |
|  | Vd | Ventricular drainage |
|  | Diuretic | Use of diuretics |
|  | β-blocker | Use of beta-blockers |
| **Outcomes** | ICU Mortality | ICU mortality |
|  | In-hospital Mortality | In-hospital mortality |
|  | 30-Day Mortality | Mortality at 30 days post-admission |
|  | 60-Day Mortality | Mortality at 60 days post-admission |
|  | 90-Day Mortality | Mortality at 90 days post-admission |
|  | 365-Day Mortality | Mortality at 365 days post-admission |

# Supplementary Table 2. Univariate Analysis of SHR and Associated Variables for In-Hospital Mortality

|  | **Before Imputation** | | **After Imputation** | |
| --- | --- | --- | --- | --- |
| **Item** | **HR (95%CI)** | **P-Value** | **HR (95%CI)** | **P-Value** |
| **SHR (continuous variable)** | 1.25 (1.17~1.34) | <0.001 | 1.25 (1.17~1.34) | <0.001 |
| **Demographic** |  |  |  |  |
| **Sex，Male** | 0.89 (0.72~1.1) | 0.29 | 0.89 (0.72~1.1) | 0.289 |
| **Age** | 1.02 (1.01~1.02) | <0.001 | 1.02 (1.01~1.02) | <0.001 |
| **Weight** | 0.99 (0.99~1) | 0.018 | 0.99 (0.99~1) | 0.016 |
| **Vital signs** |  |  |  |  |
| **Heart rate** | 1.01 (1.01~1.02) | <0.001 | 1.01 (1.01~1.02) | <0.001 |
| **MBP** | 0.97 (0.96~0.98) | <0.001 | 0.97 (0.96~0.98) | <0.001 |
| **Respiratory rate** | 1.08 (1.05~1.12) | <0.001 | 1.08 (1.05~1.11) | <0.001 |
| **Temperature** | 1.14 (0.89~1.45) | 0.313 | 1.14 (0.89~1.46) | 0.293 |
| **Spo2** | 1.21 (1.14~1.29) | <0.001 | 1.21 (1.14~1.29) | <0.001 |
| **Laboratory tests** | |  |  |  |
| **Hemoglobin** | 0.89 (0.85~0.93) | <0.001 | 0.89 (0.85~0.93) | <0.001 |
| **Platelets** | 1 (1~1) | <0.001 | 1 (1~1) | <0.001 |
| **RBC** | 0.79 (0.71~0.87) | <0.001 | 0.79 (0.71~0.87) | <0.001 |
| **WBC** | 1.01 (1.01~1.02) | <0.001 | 1.01 (1.01~1.02) | <0.001 |
| **Urea nitrogen** | 1.01 (1.01~1.02) | <0.001 | 1.01 (1.01~1.02) | <0.001 |
| **Creatinine** | 1.14 (1.08~1.21) | <0.001 | 1.14 (1.08~1.21) | <0.001 |
| **Sodium** | 1.02 (1~1.05) | 0.072 | 1.02 (1~1.05) | 0.08 |
| **Potassium** | 0.94 (0.77~1.15) | 0.562 | 0.94 (0.77~1.16) | 0.579 |
| **AST** | 1 (1~1) | 0.317 | 1 (1~1) | 0.637 |
| **Medical history** |  |  |  |  |
| **Smoke** | 1.09 (0.87~1.36) | 0.473 | 1.09 (0.87~1.36) | 0.472 |
| **Organ dysfunction** | |  |  |  |
| **Dementia** | 1.28 (0.88~1.85) | 0.193 | 1.28 (0.88~1.85) | 0.192 |
| **CBD** | 0.79 (0.5~1.23) | 0.295 | 0.79 (0.5~1.23) | 0.294 |
| **Cancer** | 1.08 (0.74~1.59) | 0.685 | 1.08 (0.74~1.59) | 0.685 |
| **Rheumatic** | 1.21 (0.6~2.44) | 0.593 | 1.21 (0.6~2.44) | 0.593 |
| **Liver disease** | 1.39 (0.96~2.01) | 0.083 | 1.39 (0.96~2.01) | 0.082 |
| **Hyperlipidemia** | 0.87 (0.7~1.08) | 0.201 | 0.87 (0.7~1.08) | 0.2 |
| **Diabetes** | 1.09 (0.88~1.36) | 0.416 | 1.09 (0.88~1.36) | 0.416 |
| **HBP** | 0.87 (0.68~1.12) | 0.284 | 0.87 (0.68~1.12) | 0.284 |
| **MI** | 1.1 (0.82~1.47) | 0.52 | 1.1 (0.82~1.47) | 0.52 |
| **CHF** | 1.21 (0.95~1.53) | 0.119 | 1.21 (0.95~1.53) | 0.118 |
| **Sepsis3** | 1.43 (1.15~1.78) | 0.002 | 1.43 (1.15~1.78) | 0.001 |
| **Score** |  |  |  |  |
| **CCI** | 1.05 (1.02~1.09) | 0.006 | 1.05 (1.02~1.09) | 0.005 |
| **GCS** | 0.91 (0.89~0.94) | <0.001 | 0.91 (0.89~0.94) | <0.001 |
| **SAPS II** | 1.04 (1.04~1.05) | <0.001 | 1.04 (1.04~1.05) | <0.001 |
| **In-hospital procedures** | |  |  |  |
| **Vent1day** | 2.53 (2.05~3.12) | <0.001 | 2.53 (2.05~3.12) | <0.001 |
| **Craniotomy** | 1.13 (0.82~1.56) | 0.466 | 1.13 (0.82~1.56) | 0.466 |
| **Pe** | 0.8 (0.59~1.09) | 0.162 | 0.8 (0.59~1.09) | 0.161 |
| **Vd** | 1.28 (0.75~2.19) | 0.368 | 1.28 (0.75~2.19) | 0.368 |
| **Diuretic** | 1.21 (0.97~1.49) | 0.087 | 1.21 (0.97~1.49) | 0.087 |
| **β_blocker** | 1 (0.78~1.28) | 0.991 | 1 (0.78~1.28) | 0.991 |

**Note:** Variables with **p < 0.1** from the univariate analysis include: Age, weight, heart rate (HR), mean blood pressure (MBP), respiratory rate (RR), SpO₂, hemoglobin, platelets, red blood cell count (RBC), white blood cell count (WBC), blood urea nitrogen (BUN), creatinine, sodium, liver disease, and Sepsis3.Additionally, clinically significant variables (despite **p > 0.1**) such as hypertension, diabetes, and craniotomy were included in the multivariable analysis due to their established clinical relevance in the prognosis of ABI patients.

# Supplementary Table 3. Association of Selected Variables with Mortality Outcomes

|  | **In-hospital Mortality** | |  |  | **ICU Mortality** | |  |  | **365-Day Mortality** | |  |  | **30-Day Mortality** | |  |  | **60-Day Mortality** | |  |  | **90-Day Mortality** | |  |  |
| --- | --- | --- | --- | --- | --- | --- | --- | --- | --- | --- | --- | --- | --- | --- | --- | --- | --- | --- | --- | --- | --- | --- | --- | --- |
|  | **Model 1** |  | **Model 3** |  | **Model 1** |  | **Model 3** |  | **Model 1** |  | **Model 3** |  | **Model 1** |  | **Model 3** |  | **Model 1** |  | **Model 3** |  | **Model 1** |  | **Model 3** |  |
| **Variable** | HR（95%CI） | P-value | HR（95%CI） | P-value | HR（95%CI） | P-value | HR（95%CI） | P-value | HR（95%CI） | P-value | HR（95%CI） | P-value | HR（95%CI） | P-value | HR（95%CI） | P-value | HR（95%CI） | P-value | HR（95%CI） | P-value | HR（95%CI） | P-value | HR（95%CI） | P-value |
| **Age** | 1.02 (1.01~1.02) | <0.001 | 1.02 (1.01~1.03) | <0.001 | 1 (1~1.01) | 0.37 | 1.02 (1~1.03) | 0.016 | 1.03 (1.03~1.04) | <0.001 | 1.02 (1.01~1.03) | <0.001 | 1.02 (1.02~1.03) | <0.001 | 1.02 (1.01~1.03) | <0.001 | 1.02 (1.02~1.03) | <0.001 | 1.02 (1.01~1.03) | <0.001 | 1.03 (1.02~1.03) | <0.001 | 1.02 (1.01~1.03) | <0.001 |
| **Weight** | 0.99 (0.99~1) | 0.016 | 1 (0.99~1) | 0.223 | 1 (0.99~1) | 0.524 | 1.01 (1~1.01) | 0.031 | 0.99 (0.99~0.99) | <0.001 | 0.99 (0.99~1) | 0.003 | 0.99 (0.99~0.99) | <0.001 | 0.99 (0.99~1) | 0.037 | 0.99 (0.98~0.99) | <0.001 | 0.99 (0.99~1) | 0.007 | 0.99 (0.99~0.99) | <0.001 | 0.99 (0.99~1) | 0.006 |
| **Heart rate** | 1.01 (1.01~1.02) | <0.001 | 1.01 (1~1.01) | 0.108 | 1.01 (1~1.02) | 0.004 | 1.01 (1~1.01) | 0.247 | 1.02 (1.02~1.03) | <0.001 | 1.01 (1.01~1.02) | <0.001 | 1.02 (1.02~1.03) | <0.001 | 1.01 (1.01~1.02) | <0.001 | 1.02 (1.02~1.03) | <0.001 | 1.01 (1.01~1.02) | <0.001 | 1.03 (1.02~1.03) | <0.001 | 1.01 (1.01~1.02) | <0.001 |
| **MBP** | 0.97 (0.96~0.98) | <0.001 | 0.99 (0.98~1) | 0.193 | 0.98 (0.97~0.99) | <0.001 | 1 (0.99~1.01) | 0.956 | 0.97 (0.97~0.98) | <0.001 | 0.99 (0.99~1) | 0.19 | 0.97 (0.96~0.98) | <0.001 | 1 (0.99~1.01) | 0.742 | 0.97 (0.96~0.98) | <0.001 | 1 (0.99~1.01) | 0.65 | 0.97 (0.97~0.98) | <0.001 | 1 (0.99~1.01) | 0.698 |
| **Respiratory rate** | 1.08 (1.05~1.11) | <0.001 | 1.09 (1.05~1.13) | <0.001 | 1.07 (1.04~1.11) | <0.001 | 1.07 (1.03~1.12) | 0.002 | 1.11 (1.09~1.13) | <0.001 | 1.07 (1.04~1.1) | <0.001 | 1.13 (1.1~1.16) | <0.001 | 1.09 (1.06~1.12) | <0.001 | 1.13 (1.1~1.16) | <0.001 | 1.09 (1.06~1.13) | <0.001 | 1.13 (1.1~1.15) | <0.001 | 1.09 (1.06~1.12) | <0.001 |
| **Spo2** | 1.21 (1.14~1.29) | <0.001 | 1.15 (1.07~1.24) | <0.001 | 1.25 (1.15~1.34) | <0.001 | 1.2 (1.1~1.32) | <0.001 | 1.19 (1.14~1.24) | <0.001 | 1.11 (1.06~1.17) | <0.001 | 1.26 (1.19~1.33) | <0.001 | 1.16 (1.09~1.24) | <0.001 | 1.24 (1.18~1.3) | <0.001 | 1.14 (1.08~1.21) | <0.001 | 1.24 (1.18~1.3) | <0.001 | 1.15 (1.08~1.21) | <0.001 |
| **Hemoglobin** | 0.89 (0.85~0.93) | <0.001 | 1.04 (0.96~1.11) | 0.344 | 0.91 (0.86~0.96) | <0.001 | 1.01 (0.93~1.1) | 0.807 | 0.83 (0.8~0.86) | <0.001 | 1 (0.96~1.06) | 0.857 | 0.84 (0.81~0.87) | <0.001 | 1.01 (0.95~1.07) | 0.767 | 0.83 (0.81~0.87) | <0.001 | 1 (0.95~1.06) | 0.922 | 0.83 (0.8~0.86) | <0.001 | 1 (0.94~1.06) | 0.93 |
| **Platelets** | 1 (1~1) | <0.001 | 1 (1~1) | 0.369 | 1 (1~1) | 0.001 | 1 (1~1) | 0.401 | 1 (1~1) | <0.001 | 1 (1~1) | 0.292 | 1 (1~1) | <0.001 | 1 (1~1) | 0.244 | 1 (1~1) | <0.001 | 1 (1~1) | 0.145 | 1 (1~1) | <0.001 | 1 (1~1) | 0.372 |
| **RBC** | 0.79 (0.71~0.87) | <0.001 | 0.89 (0.74~1.06) | 0.194 | 0.82 (0.73~0.92) | 0.001 | 0.93 (0.75~1.16) | 0.518 | 0.78 (0.73~0.82) | <0.001 | 0.91 (0.81~1.02) | 0.089 | 0.78 (0.72~0.84) | <0.001 | 0.9 (0.78~1.05) | 0.176 | 0.78 (0.73~0.84) | <0.001 | 0.91 (0.79~1.05) | 0.214 | 0.79 (0.74~0.84) | <0.001 | 0.93 (0.81~1.07) | 0.313 |
| **WBC** | 1.01 (1.01~1.02) | <0.001 | 1.01 (1.01~1.02) | <0.001 | 1.01 (1.01~1.02) | <0.001 | 1.01 (1~1.02) | 0.009 | 1.02 (1.01~1.02) | <0.001 | 1.01 (1~1.01) | 0.008 | 1.02 (1.01~1.02) | <0.001 | 1.01 (1.01~1.02) | <0.001 | 1.02 (1.01~1.02) | <0.001 | 1.01 (1~1.02) | <0.001 | 1.02 (1.01~1.02) | <0.001 | 1.01 (1~1.01) | 0.001 |
| **Urea nitrogen** | 1.01 (1.01~1.02) | <0.001 | 1 (0.99~1.01) | 0.643 | 1.01 (1~1.01) | 0.035 | 0.99 (0.98~1) | 0.045 | 1.02 (1.02~1.02) | <0.001 | 1 (0.99~1.01) | 0.956 | 1.02 (1.01~1.02) | <0.001 | 1 (0.99~1) | 0.647 | 1.02 (1.01~1.02) | <0.001 | 1 (0.99~1) | 0.527 | 1.02 (1.02~1.02) | <0.001 | 1 (0.99~1) | 0.725 |
| **Creatinine** | 1.14 (1.08~1.21) | <0.001 | 1.11 (1.01~1.21) | 0.031 | 1.08 (1~1.18) | 0.058 | 1.07 (0.95~1.2) | 0.285 | 1.18 (1.12~1.23) | <0.001 | 1.06 (0.98~1.15) | 0.116 | 1.15 (1.09~1.22) | <0.001 | 1.04 (0.95~1.15) | 0.393 | 1.16 (1.1~1.22) | <0.001 | 1.07 (0.98~1.17) | 0.113 | 1.17 (1.11~1.23) | <0.001 | 1.07 (0.98~1.16) | 0.131 |
| **Sodium** | 1.02 (1~1.05) | 0.08 | 1.03 (1~1.05) | 0.021 | 1.02 (0.99~1.05) | 0.16 | 1.02 (1~1.05) | 0.06 | 1 (0.98~1.02) | 0.936 | 1.01 (1~1.03) | 0.162 | 1 (0.98~1.03) | 0.654 | 1.02 (1~1.04) | 0.048 | 1.01 (0.99~1.03) | 0.411 | 1.02 (1~1.04) | 0.023 | 1 (0.99~1.02) | 0.659 | 1.02 (1~1.03) | 0.069 |
| **Liver disease** | 1.39 (0.96~2.01) | 0.082 | 1.19 (0.78~1.83) | 0.416 | 1.28 (0.81~2) | 0.29 | 1.09 (0.64~1.86) | 0.741 | 1.87 (1.41~2.48) | <0.001 | 1.23 (0.9~1.68) | 0.199 | 1.85 (1.32~2.6) | <0.001 | 1.16 (0.79~1.7) | 0.443 | 1.85 (1.34~2.56) | <0.001 | 1.16 (0.81~1.67) | 0.42 | 1.94 (1.43~2.63) | <0.001 | 1.23 (0.88~1.74) | 0.228 |
| **Diabetes** | 0.87 (0.68~1.12) | 0.284 | 0.75 (0.57~0.99) | 0.042 | 0.63 (0.48~0.84) | 0.001 | 0.63 (0.45~0.88) | 0.006 | 1.1 (0.92~1.32) | 0.302 | 0.81 (0.67~0.99) | 0.037 | 0.97 (0.78~1.21) | 0.78 | 0.8 (0.63~1.01) | 0.061 | 0.99 (0.81~1.22) | 0.944 | 0.79 (0.63~0.99) | 0.045 | 0.99 (0.81~1.21) | 0.927 | 0.77 (0.62~0.96) | 0.019 |
| **HBP** | 1.09 (0.88~1.36) | 0.416 | 1.1 (0.86~1.4) | 0.456 | 0.9 (0.68~1.18) | 0.433 | 0.99 (0.73~1.34) | 0.94 | 1.17 (1.01~1.36) | 0.041 | 0.92 (0.78~1.08) | 0.3 | 1.04 (0.86~1.26) | 0.688 | 0.91 (0.74~1.12) | 0.383 | 1.02 (0.86~1.22) | 0.8 | 0.88 (0.72~1.07) | 0.188 | 1.06 (0.89~1.26) | 0.504 | 0.87 (0.72~1.06) | 0.161 |
| **Sepsis3** | 1.43 (1.15~1.78) | 0.001 | 0.76 (0.58~0.99) | 0.045 | 1.17 (0.88~1.57) | 0.276 | 0.68 (0.48~0.96) | 0.031 | 2.42 (2.09~2.79) | <0.001 | 1.17 (0.98~1.41) | 0.089 | 2.58 (2.15~3.08) | <0.001 | 1.11 (0.88~1.39) | 0.389 | 2.54 (2.15~3) | <0.001 | 1.08 (0.87~1.34) | 0.471 | 2.57 (2.19~3.02) | <0.001 | 1.11 (0.9~1.36) | 0.321 |
| **CCI** | 1.05 (1.02~1.09) | 0.005 | 0.97 (0.92~1.03) | 0.32 | 0.98 (0.94~1.03) | 0.464 | 0.93 (0.87~1) | 0.057 | 1.19 (1.16~1.22) | <0.001 | 1.09 (1.05~1.13) | <0.001 | 1.12 (1.09~1.15) | <0.001 | 1.03 (0.98~1.07) | 0.282 | 1.13 (1.1~1.17) | <0.001 | 1.04 (0.99~1.08) | 0.112 | 1.15 (1.12~1.18) | <0.001 | 1.05 (1.01~1.1) | 0.016 |
| **GCS** | 0.91 (0.89~0.94) | <0.001 | 0.98 (0.95~1.02) | 0.332 | 0.96 (0.92~0.99) | 0.017 | 1.03 (0.99~1.08) | 0.113 | 0.85 (0.83~0.87) | <0.001 | 0.93 (0.9~0.95) | <0.001 | 0.84 (0.82~0.86) | <0.001 | 0.94 (0.91~0.97) | <0.001 | 0.84 (0.82~0.86) | <0.001 | 0.93 (0.9~0.96) | <0.001 | 0.84 (0.82~0.86) | <0.001 | 0.93 (0.9~0.96) | <0.001 |
| **SAPS II** | 1.04 (1.04~1.05) | <0.001 | 1.02 (1.01~1.03) | 0.001 | 1.04 (1.03~1.05) | <0.001 | 1.04 (1.03~1.05) | <0.001 | 1.06 (1.06~1.07) | <0.001 | 1.02 (1.01~1.03) | <0.001 | 1.06 (1.05~1.07) | <0.001 | 1.02 (1.01~1.03) | <0.001 | 1.06 (1.05~1.07) | <0.001 | 1.02 (1.01~1.03) | <0.001 | 1.06 (1.06~1.07) | <0.001 | 1.02 (1.01~1.03) | <0.001 |
| **Vent1day** | 2.53 (2.05~3.12) | <0.001 | 1.94 (1.49~2.52) | <0.001 | 2.56 (1.98~3.32) | <0.001 | 1.89 (1.39~2.58) | <0.001 | 2.19 (1.88~2.55) | <0.001 | 1.23 (1.02~1.49) | 0.03 | 3.04 (2.54~3.64) | <0.001 | 1.71 (1.36~2.14) | <0.001 | 2.79 (2.35~3.31) | <0.001 | 1.56 (1.26~1.94) | <0.001 | 2.64 (2.24~3.12) | <0.001 | 1.47 (1.2~1.82) | <0.001 |
| **Craniotomy** | 1.13 (0.82~1.56) | 0.466 | 1.13 (0.81~1.59) | 0.471 | 1.15 (0.82~1.63) | 0.419 | 1.05 (0.73~1.52) | 0.787 | 1.43 (1.11~1.83) | 0.005 | 1.32 (1.02~1.72) | 0.038 | 1.54 (1.14~2.06) | 0.004 | 1.22 (0.89~1.67) | 0.222 | 1.48 (1.11~1.96) | 0.007 | 1.22 (0.9~1.65) | 0.203 | 1.4 (1.06~1.85) | 0.017 | 1.18 (0.87~1.58) | 0.283 |
| **Diuretic** | 1.21 (0.97~1.49) | 0.087 | 0.81 (0.63~1.04) | 0.092 | 0.72 (0.55~0.93) | 0.014 | 0.57 (0.42~0.78) | <0.001 | 1.74 (1.51~2.01) | <0.001 | 0.9 (0.76~1.07) | 0.253 | 1.68 (1.41~2.01) | <0.001 | 0.83 (0.67~1.02) | 0.081 | 1.74 (1.47~2.06) | <0.001 | 0.89 (0.73~1.08) | 0.242 | 1.82 (1.55~2.14) | <0.001 | 0.92 (0.76~1.11) | 0.391 |

**Notes:** This table presents the association of 23 selected variables with mortality outcomes, including in-hospital mortality, ICU mortality, 30-day, 60-day, 90-day, and 365-day mortality, using two models: Model 1 (unadjusted) and Model 3 (adjusted for covariates). The variables were selected based on univariate analysis (p < 0.1) and their clinical relevance, with 14 of these variables also identified through stepwise regression. Model 3 adjusts for admission age, weight, heart rate, mean blood pressure (MBP), respiratory rate, SpO₂, hemoglobin, platelets, red blood cells (RBC), white blood cells (WBC), urea nitrogen (BUN), creatinine, sodium, liver disease, diabetes, hypertension (HBP), Sepsis3, Charlson comorbidity index (CCI), Glasgow coma scale (GCS), SAPS II, ventilation on the first ICU day, craniotomy, diuretic use, and SHR. Hazard ratios (HR) with 95% confidence intervals (CI) and P-values are presented for each variable in relation to the different mortality outcomes.

# Supplementary Table 4. Predictors of In-Hospital Mortality Identified by Stepwise Regression.

| **Variable** | **Estimate** | **HR** | **Std.Error** | **z.value** | **P.value** | **AIC** |
| --- | --- | --- | --- | --- | --- | --- |
| **Age** | 0.016 | 1.016 | 0.004 | 3.499 | <0.001 | 4765.846 |
| **Heart rate** | 0.006 | 1.006 | 0.004 | 1.666 | 0.096 |  |
| **MBP** | -0.009 | 0.991 | 0.005 | -1.63 | 0.103 |  |
| **Respiratory rate** | 0.088 | 1.091 | 0.018 | 4.877 | <0.001 |  |
| **Spo2** | 0.147 | 1.158 | 0.035 | 4.176 | <0.001 |  |
| **RBC** | -0.092 | 0.912 | 0.062 | -1.471 | 0.141 |  |
| **WBC** | 0.011 | 1.011 | 0.003 | 3.604 | <0.001 |  |
| **Creatinine** | 0.072 | 1.075 | 0.038 | 1.889 | 0.059 |  |
| **Sodium** | 0.021 | 1.021 | 0.011 | 1.959 | 0.05 |  |
| **HBP** | -0.265 | 0.768 | 0.138 | -1.92 | 0.055 |  |
| **Sepsis3** | -0.242 | 0.785 | 0.133 | -1.824 | 0.068 |  |
| **SAPS II** | 0.023 | 1.024 | 0.005 | 4.508 | <0.001 |  |
| **Vent1day** | 0.714 | 2.042 | 0.128 | 5.587 | <0.001 |  |
| **Diuretic** | -0.242 | 0.785 | 0.123 | -1.972 | 0.049 |  |

**Note:** This table presents the results of stepwise regression analysis identifying predictors of in-hospital mortality. Model selection was based on minimizing the Akaike Information Criterion (AIC). The table includes regression coefficients (Estimate), hazard ratios (HR), standard errors (Std. Error), Z-values, P-values, and AIC values for model comparison. Variables retained in the final model include Age, Heart Rate, Mean Blood Pressure (MBP), Respiratory Rate, SpO₂, Red Blood Cells (RBC), White Blood Cells (WBC), Creatinine, Sodium, Hypertension (HBP), Sepsis3, SAPS II, Vent1day, and Diuretic use.

# Supplementary Table 5. Association of SHR with Mortality Outcomes Adjusted for Stepwise Regression Selected Covariates

|  | **In-hospital Mortality** | | **ICU Mortality** | | **365-Day Mortality** | | **30-Day Mortality** | | **60-Day Mortality** |  | **90-Day Mortality** | |
| --- | --- | --- | --- | --- | --- | --- | --- | --- | --- | --- | --- | --- |
| **Categories** | **HR（95%CI）** | **P-value** | **HR（95%CI）** | **P-value** | **HR（95%CI）** | **P-value** | **HR（95%CI）** | **P-value** | **HR（95%CI）** | **P-value** | **HR（95%CI）** | **P-value** |
| **SHR (continuous variable)** | 1.18 (1.06~1.31) | 0.003 | 1.12 (0.98~1.27) | 0.088 | 1.13 (1.04~1.22) | 0.004 | 1.16 (1.06~1.26) | 0.001 | 1.15 (1.06~1.25) | 0.001 | 1.14 (1.05~1.24) | 0.002 |
| **SHR (Dichotomized)** |  |  |  |  |  |  |  |  |  |  |  |  |
| **Group1** | Ref | Ref | Ref | Ref | Ref | Ref | Ref | Ref | Ref | Ref | Ref | Ref |
| **Group2** | 1.44 (1.14~1.82) | 0.003 | 1.47 (1.09~1.98) | 0.013 | 1.34 (1.14~1.56) | <0.001 | 1.6 (1.31~1.95) | <0.001 | 1.49 (1.24~1.79) | <0.001 | 1.42 (1.2~1.7) | <0.001 |

**Notes:** This table shows the association between SHR and mortality outcomes, including in-hospital and ICU mortality as short-term indicators, and 365-day mortality as a long-term indicator. Additionally, 30-day, 60-day, and 90-day mortality were assessed as secondary short-term outcomes. All results are adjusted for covariates selected through stepwise regression, including Age, Heart Rate, MBP, Respiratory Rate, SpO₂, RBC, WBC, Creatinine, Sodium, Hypertension (HBP), Sepsis3, SAPS II, Vent1day, and Diuretic use. SHR is presented as both a continuous and dichotomized variable (Group 2 vs. Group 1). Hazard ratios (HR) with 95% confidence intervals (CI) and P-values are provided for each outcome.

# Supplementary Fig.1 Flowchart illustrating patient enrollment throughout the study. MIMIC-IV: Medical Information Mart for Intensive Care-IV; ABI: acute brain injury; ICU: intensive care unit.


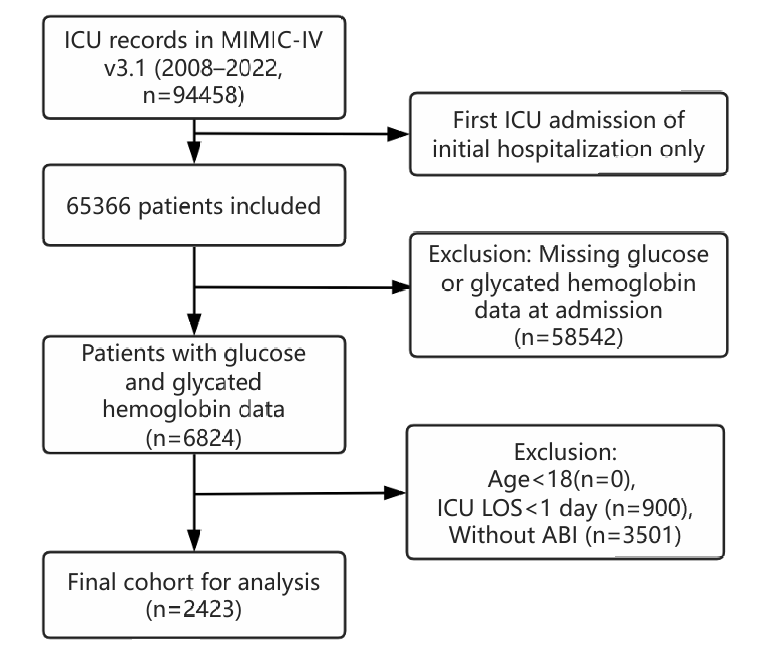


# Supplementary Fig.2 Proportional Hazards Assumption Test. Log-log survival plots and Schoenfeld residuals were used to test the proportional hazards assumption. The dashed red lines represent the time-varying hazard, the solid blue line shows the average hazard, and the dotted line indicates the reference for null effect.


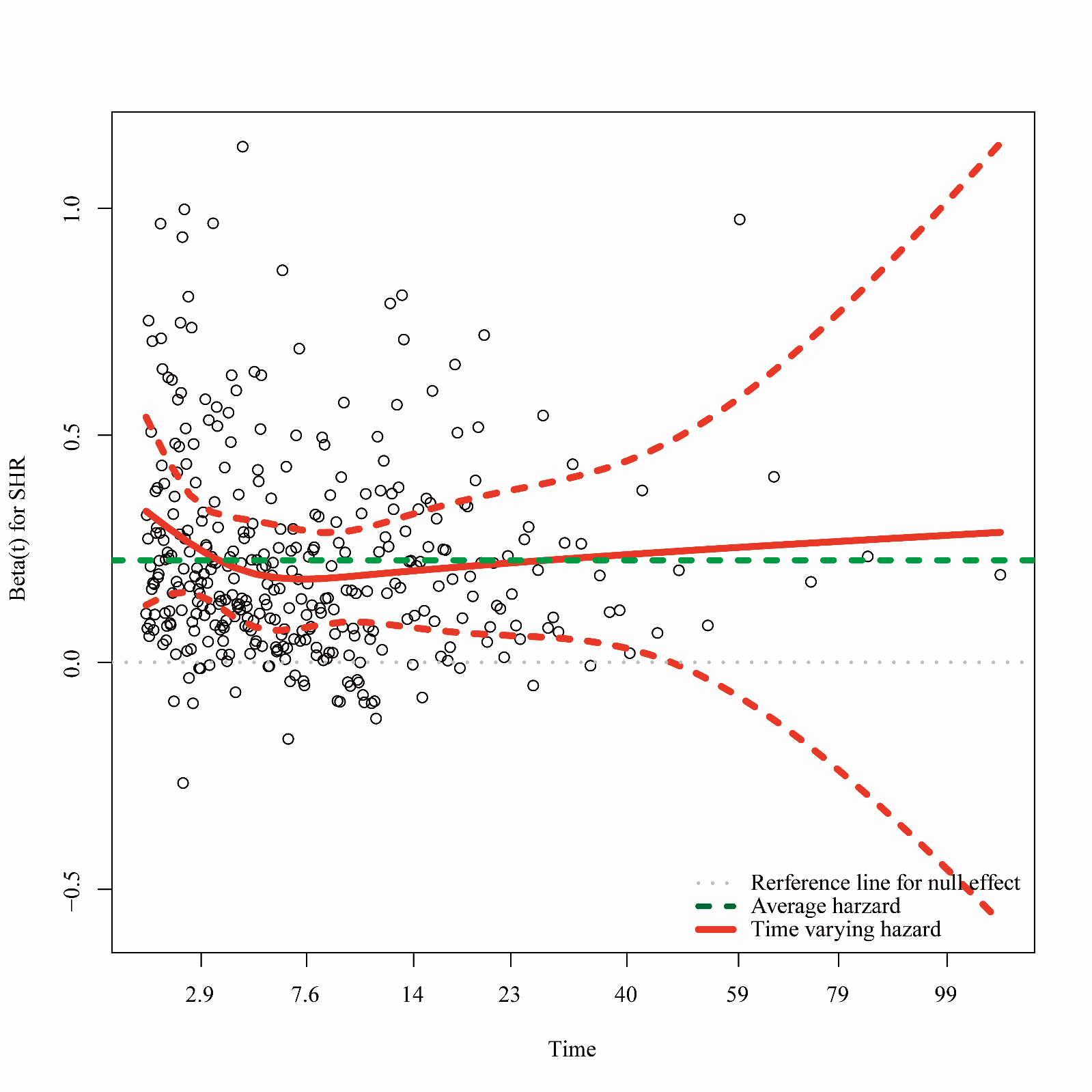


# **Supplementary Fig.3 Kaplan-Meier Survival Curves for Secondary Mortality Outcomes Stratified by SHR Groups in Patients with Acute Brain Injury. (A) 30-Day mortality, (B) 60-Day Mortality, (C) 90-Day Mortality. Notes: Kaplan–Meier curves display survival probability (%) over time for two SHR groups (Group 1 and Group 2). P-values indicate the statistical significance of survival differences between the groups. Insets show magnified views of early survival differences. Number at risk tables below each curve indicate the number of participants remaining in each group over time.**


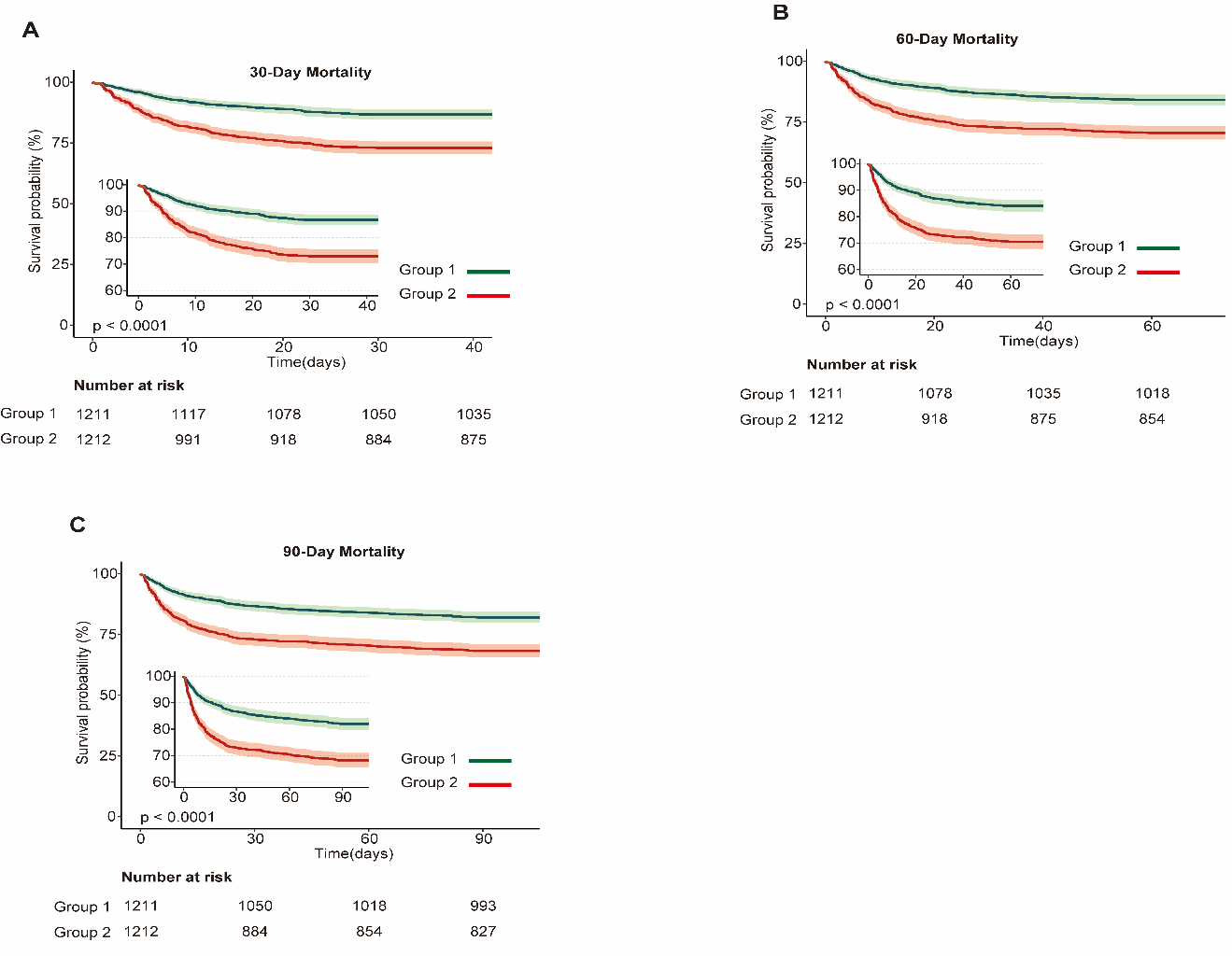


# **Supplementary** Fig.4 Restricted cubic spline curves showing the association between SHR and secondary mortality outcomes: (A) 30-Day Mortality, (B)60-Day Mortality and (C) 90-Day Mortality. Abbreviations: SHR, stress hyperglycemia ratio; HR, hazard ratio; CI, confidence interval. Notes: The red lines represent the fitted hazard ratios with 95% confidence intervals (yellow shading) based on restricted cubic spline models. The y-axis shows the hazard ratio on a logarithmic scale, while the x-axis represents SHR values. The histograms at the bottom of each plot display the distribution of SHR values in the study population. P for non-linearity indicates the statistical significance of the non-linear relationship between SHR and each mortality outcome.


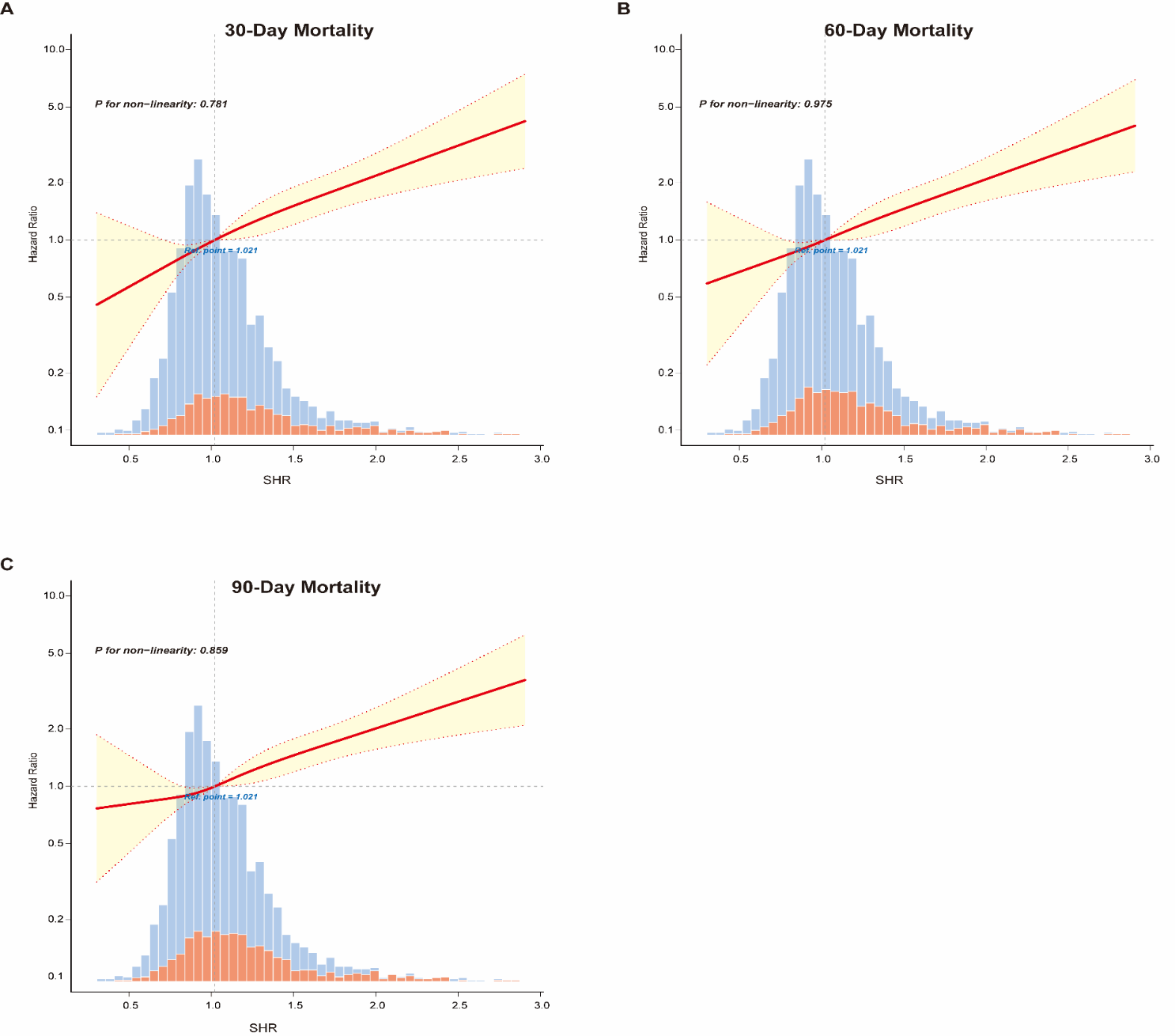


# **Supplementary** Fig.5 Receiver Operating Characteristic (ROC) Curves for Predictive Models of Mortality in ABI Patients. (A) ROC curve for 60-day mortality. (B) ROC curve for 90-day mortality.


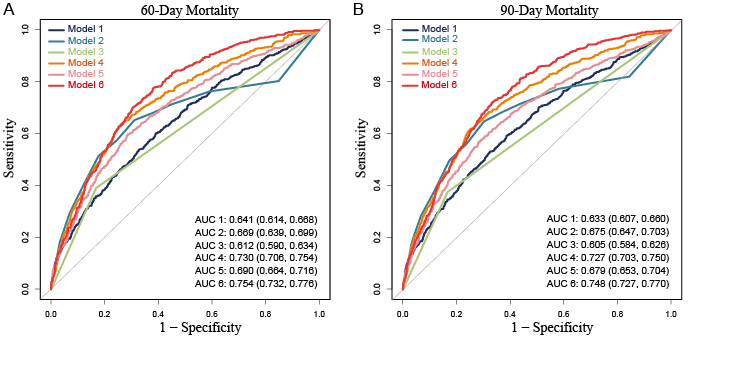


# **Supplementary** Fig.6 Variable selection and model comparison. (A) LASSO coefficient path showing the selected variables SAPS II and Vent1day. (B) Partial likelihood deviance plot for LASSO regression, with the optimal Lambda value indicated. (C) ROC curve comparison between Model_LASSO (blue), built with SAPS II and Vent1day, and Model_SHR (red), incorporating SHR, SAPS II, and Vent1day, showing AUC values of 0.773 and 0.798, respectively (P < 0.001). (D) ROC curve comparison between Model_Base (blue), built with GCS, SAPS II, and Vent1day, and Model_Full (red), incorporating GCS, SAPS II, Vent1day, and SHR, showing AUC values of 0.790 and 0.810, respectively (P < 0.001).


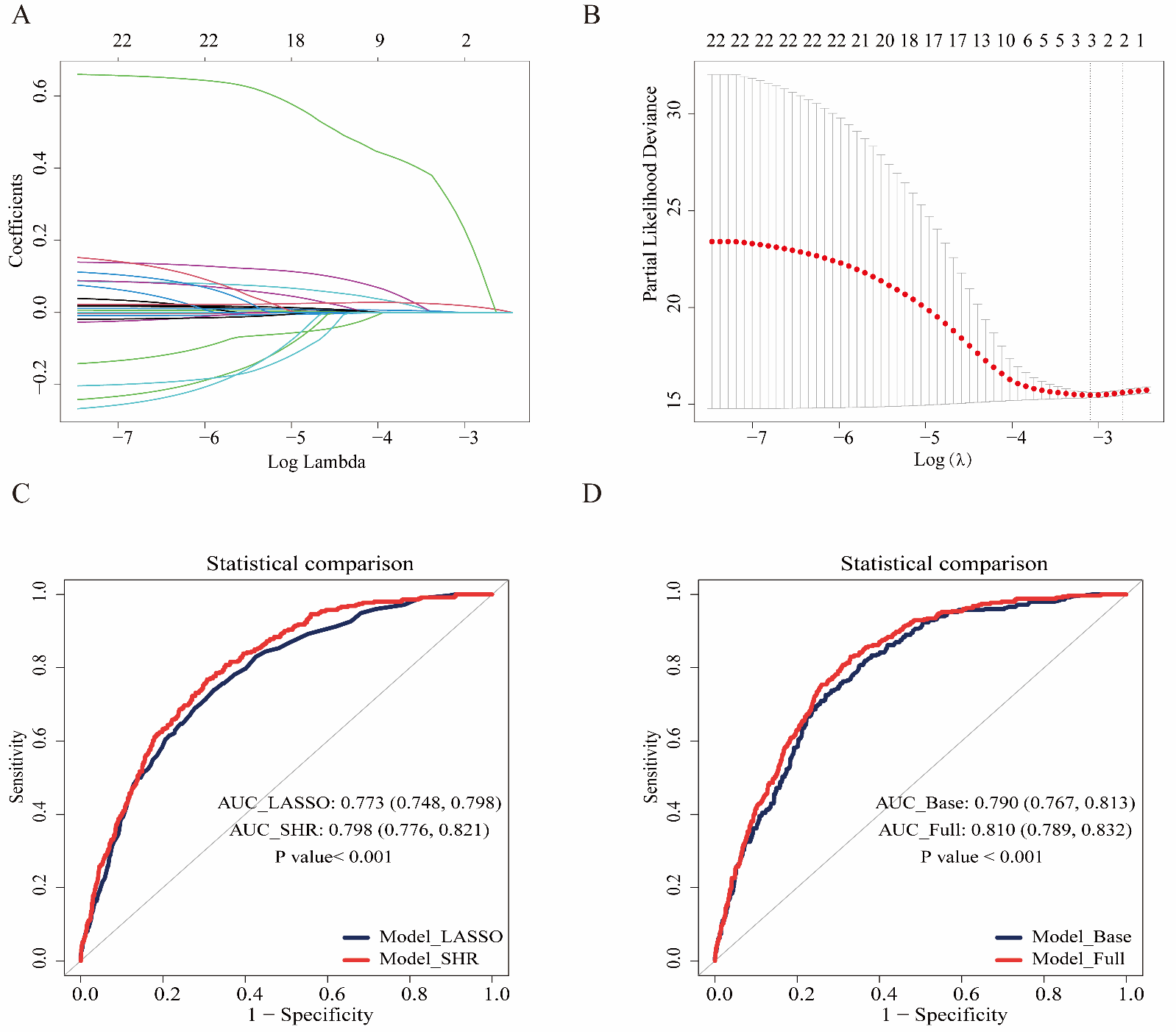


# **Supplementary** Fig.7 Time-Dependent AUC for Predicting in-hospital Mortality in Acute Brain Injury (ABI) Patients. This figure shows the time-dependent area under the curve (AUC) for Model_Base (blue), built with GCS and Vent1day, and Model_SHR (red), incorporating GCS, Vent1day, and SHR, over a 25-day period. The AUC(t) values indicate the discriminatory ability of each model at different time points, with Model_SHR consistently demonstrating higher AUC compared to Model_Base, reflecting the added predictive value of SHR in assessing mortality risk over time.


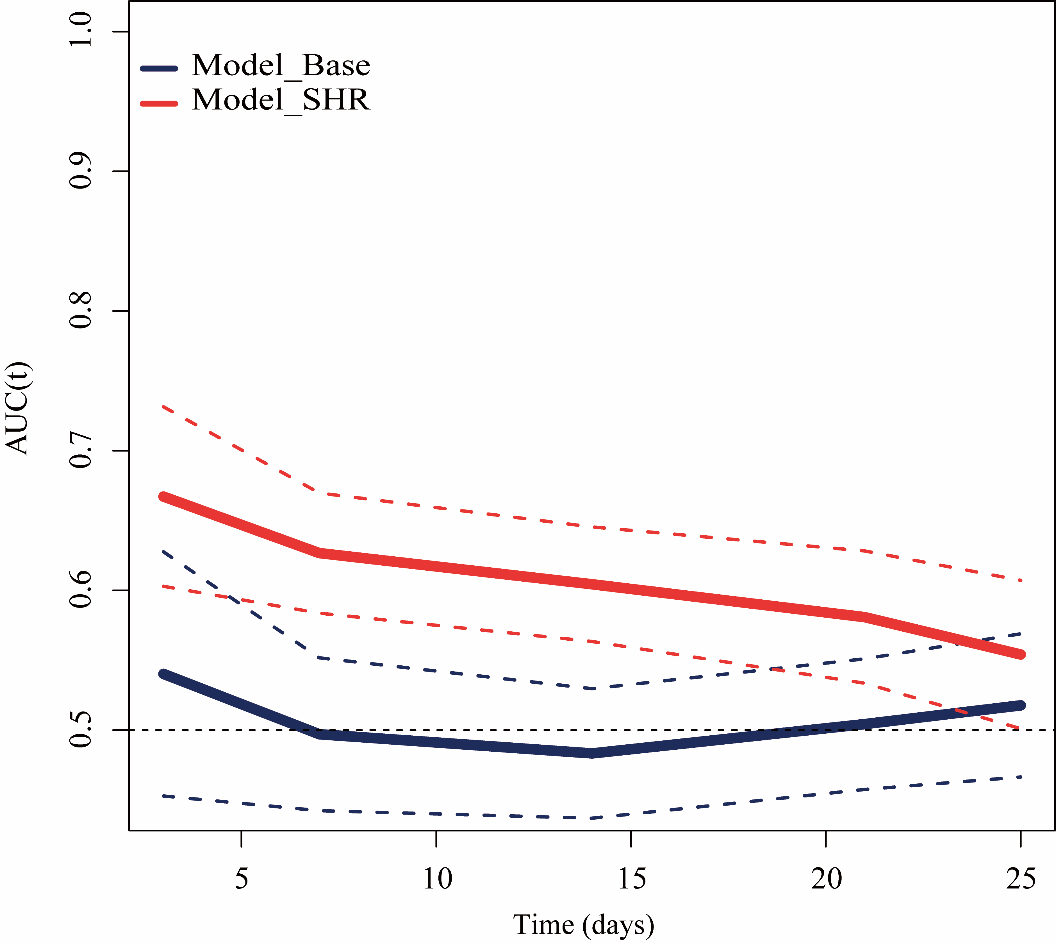


# **Supplementary** Fig.8 Bias Plots of Confounding Relative Risks for SHR as a Predictor of Mortality Outcomes in ICU Patients. (A) In-hospital mortality, (B) ICU mortality, (C) 365-day mortality, and (D) 30-day mortality, (E) 60-Day Mortality with an E-Value of 2.34 (Lower CI Bound: 1.76). (F) 90-Day Mortality with an E-Value of 2.21 (Lower CI Bound: 1.69). Abbreviations: ABI, acute brain injury; ICU, intensive care unit; HR, hazard ratio; CI, confidence interval; RREU, risk ratio for the exposure-confounder relationship; RRUD, risk ratio for the confounder-outcome relationship; RR, observed risk ratio for the exposure-outcome relationship.


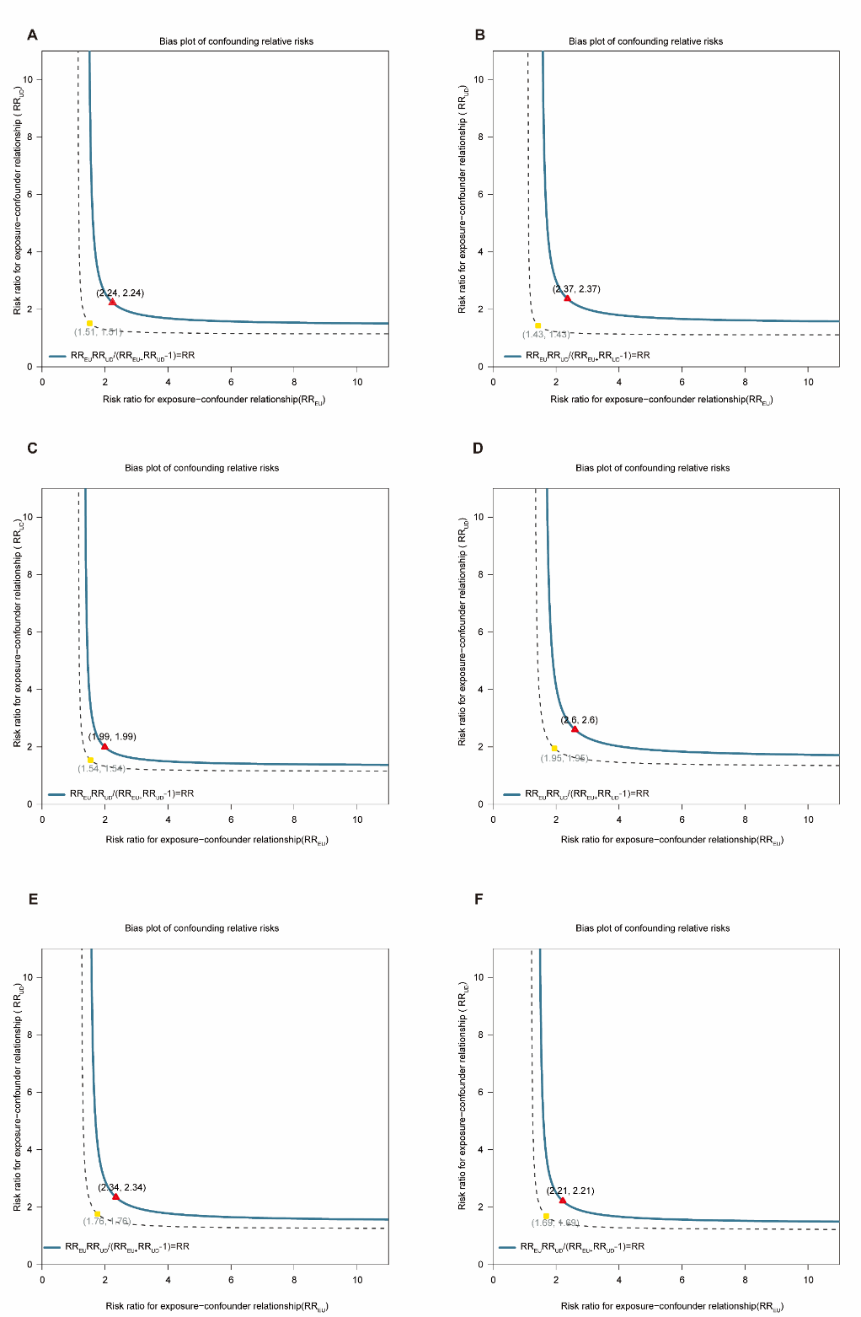

Supplement: Supplementary file 1 [file Supplementary_file_1.docx]
